# Novel Leech Antimicrobial Peptides, Hirunipins: Real‐Time 3D Monitoring of Antimicrobial and Antibiofilm Mechanisms Using Optical Diffraction Tomography

**DOI:** 10.1002/advs.202409803

**Published:** 2025-01-10

**Authors:** S. Dinesh Kumar, Jeongwon Park, Naveen Kumar Radhakrishnan, Yam Prasad Aryal, Geon‐Hwi Jeong, In‐Hyeok Pyo, Byambasuren Ganbaatar, Chul Won Lee, Sungtae Yang, Younhee Shin, Sathiyamoorthy Subramaniyam, Yu‐jin Lim, Sung‐Hak Kim, Seongsoo Lee, Song Yub Shin, Sung‐Jin Cho

**Affiliations:** ^1^ Department of Cellular & Molecular Medicine School of Medicine Chosun University Gwangju 61452 Republic of Korea; ^2^ Gwangju Center Korea Basic Science Institute (KBSI) Gwangju 61751 Republic of Korea; ^3^ Department of Animal Science Chonnam National University Gwangju 61186 South Korea; ^4^ Department of Biomedical Sciences Graduate School Chosun University Gwangju 61452 Republic of Korea; ^5^ Department of Biological Sciences and Biotechnology College of Natural Sciences Chungbuk National University Cheongju Chungbuk 28644 Republic of Korea; ^6^ Department of Chemistry Chonnam National University Gwangju 61186 Republic of Korea; ^7^ Institute of Well‐Aging Medicare & CSU G‐LAMP Project Group Chosun University Gwangju 61452 Republic of Korea; ^8^ Research and Development Center Insilicogen Inc Yongin‐si Gyeonggi‐do 16954 Republic of Korea; ^9^ Department of Bio‐Analysis Science University of Science & Technology Daejeon 34113 Republic of Korea; ^10^ Department of Systems Biotechnology Chung‐Ang University Anseong 17546 Republic of Korea; ^11^ Department of Life Science Hanyang University Seoul 04763 Republic of Korea

**Keywords:** antibiofilm, antimicrobial peptide, Hirunipin, Leech, ODT technology

## Abstract

Antimicrobial peptides (AMPs) are promising agents for treating antibiotic‐resistant bacterial infections. Although discovering novel AMPs is crucial for combating multidrug‐resistant bacteria and biofilm‐related infections, their clinical potential relies on precise, real‐time evaluation of efficacy, toxicity, and mechanisms. Optical diffraction tomography (ODT), a label‐free imaging technology, enables real‐time visualization of bacterial morphological changes, membrane damage, and biofilm formation over time. Here, a computational analysis of the leech transcriptome using an advanced AI‐based peptide screening strategy with ODT to identify potential AMPs is employed. Among the 19 potential AMPs identified, hirunipin 2 demonstrates potent antibacterial activity, low mammalian cytotoxicity, and minimal hemolytic effects. It demonstrates efficacy comparable to melittin, resistance to physiological salts and human serum, and a low likelihood of inducing bacterial resistance. Microscopy and 3D‐ODT confirm its disruption of bacterial membranes and intracellular aggregation, leading to cell death. Notably, hirunipin 2 effectively inhibits biofilm formation, eradicates preformed biofilms, and synergizes with antibiotics against multidrug‐resistant Acinetobacter baumannii (MDRAB) by enhancing membrane permeability. Additionally, hirunipin 2 significantly suppresses pro‐inflammatory cytokine expression in LPS‐stimulated macrophages, highlighting its anti‐inflammatory properties. These findings highlight hirunipin 2 as a strong candidate for developing novel antibacterial, anti‐inflammatory, and antibiofilm therapies, particularly against multidrug‐resistant bacterial infections.

## Graphical abstract

Novel antimicrobial peptides (AMPs) are identified from the medicinal leech *Hirudo nipponia* using transcriptome analysis, AI‐based in silico prediction, and optical diffraction tomography screening. Among the 19 potential AMPs identified, hirunipin 2 demonstrates potent antibacterial activity, antibiofilm and anti‐inflammatory activities. This peptide enhances the efficacy of conventional antibiotics synergistically, making it a promising therapeutic candidate.

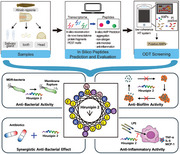

## 1 Introduction

The World Health Organization (WHO) has highlighted the critical lack of new antibiotics and the growing threat of antibiotic resistance. In their annual pipeline report, the WHO described the clinical and preclinical development of antibacterial treatments as stagnant and insufficient to meet global needs. These challenges are particularly concerning given the emergence of multidrug‐resistant (MDR) bacteria.^[^ ^1^ ^]^ ESKAPE pathogens such as *Enterococcus faecium, Staphylococcus aureus, Klebsiella pneumoniae, Acinetobacter baumannii, Pseudomonas aeruginosa*, and *Enterobacter* species are commonly associated with biofilm‐mediated wound infections, including combat wounds, blast wounds, burns, and chronic nonhealing ulcers.^[^ ^2^, ^3^ ^]^ Biofilms, which are sticky extracellular substances produced by bacteria, allow bacteria to evade antibiotics and the immune system, thereby exacerbating wound infections.^[^ ^4^ ^]^ These pathogens often exhibit multidrug resistance, which further complicates their treatment. *Acinetobacter baumannii* is a major concern due to its high multidrug resistance.^[^ ^5^ ^]^ Innovative approaches are required to address this issue. Antimicrobial peptides (AMPs) are promising sources of potent antibiotic and antibiofilm compounds.^[^ ^6^, ^7^, ^8^, ^9^ ^]^ Discovering new AMPs that effectively target biofilms is essential for combating MDR bacteria, particularly those responsible for wound infections characterized by extensive biofilm formation.

AMPs are essential and universal components of innate immunity, acting as effective weapons against various pathogens, including bacteria, fungi, viruses, and protozoa.^[^ ^10^, ^11^, ^12^ ^]^ AMPs kill pathogens by disrupting their cell membranes and employing a nonspecific mechanism that reduces the likelihood of developing antibiotic resistance.^[^ ^12^ ^]^ Leeches have gained attention as a source of AMPs for medical applications because of their remarkable ability to store ingested blood for extended periods without degradation.^[^ ^13^, ^14^ ^]^ Their immune system has evolved to inhibit the growth of external pathogens that they consume along with their food and to manage the microorganisms in their digestive tract.^[^ ^13^, ^14^ ^]^ It is evident that leeches utilize AMPs during feeding and blood storage, and these peptides must have low toxicity to blood cells to prevent excessive hemolysis. Therefore, leech‐derived AMPs are expected to be promising antimicrobial agents with low toxicity

Notable AMPs from various leech species, such as theromyzin, theromacin, neuromacin, and hydramacin‐1, exhibit strong antimicrobial activity against both gram‐positive and gram‐negative bacteria.^[^ ^15^, ^16^, ^17^, ^18^, ^19^ ^]^ However, these peptides currently face critical challenges in therapeutic development, including a limited activity spectrum and susceptibility to proteolytic degradation. This necessitates the discovery of new AMPs with enhanced properties including broader antimicrobial activity and higher stability. Newly discovered leech AMPs from leeches can be employed to predict and evaluate a variety of characteristics, including antimicrobial efficacy, physicochemical properties, and immunological toxicity. Recently, these factors have been predicted to discover rational AMPs using in silico screening tools that utilize AI‐based analysis and an extensive database of potential AMP candidates.^[^ ^20^, ^21^ ^]^ Antimicrobial and antibiofilm effects have mainly been confirmed through fluorescence (FL) imaging techniques such as cell viability assays or several other FL‐based assays. However, FL imaging has several disadvantages, particularly those associated with fixation, staining, and photobleaching. Label‐free imaging techniques, such as confocal reflectance microscopy, raman spectroscopy, and optical diffraction tomography (ODT), offer the advantage of observing live cells without the need for FL dyes or chemical stains. Among these, ODT stands out due to its ability to provide quantitative, 3D refractive index (RI) maps of individual cells, offering detailed structural and physical property data such as volume and dry mass. Real‐time monitoring of AMP efficacy using ODT allows detailed observation and analysis of individual bacterial cells, capturing morphological and functional changes after AMP treatment.^[^ ^22^ ^]^ With recent advancements in label‐free ODT imaging techniques, it may be possible to simultaneously compare the antimicrobial mechanisms and efficacies of several AMPs using screening methods. Taken together, ODT for high‐throughput screening (ODT‐HTS) may facilitate the rapid analysis of large bacterial populations, providing an effective tool for evaluating the efficacy of antimicrobial and antibiofilm effects.

Here, we performed a simultaneous screening of 19 AMP candidates derived from the transcriptome genome database of the salivary glands of the East Asian medicinal leech *Hirudo nipponia*, through in silico AMP prediction. Next, to select hirunipins 1, 2, and 3 as novel AMP candidates, we performed ODT‐HTS to validate the efficacy of the AMPs in multiple bacterial cells. We confirmed that hirunipin 2 has the potential to be an effective AMP with antimicrobial and antibiofilm properties. Real‐time monitoring of AMP efficacy using ODT‐HTS is an effective platform for studying the effects of AMPs on multiple bacterial cells and alterations in biofilm dynamics. We suggest that real‐time ODT‐HTS is a potential strategy as an effective platform for AMP evaluation against MDR and infectious diseases.

## 2 Results

### 2.1 Sequencing and de novo Transcriptome Assembly

**Figure 1:**
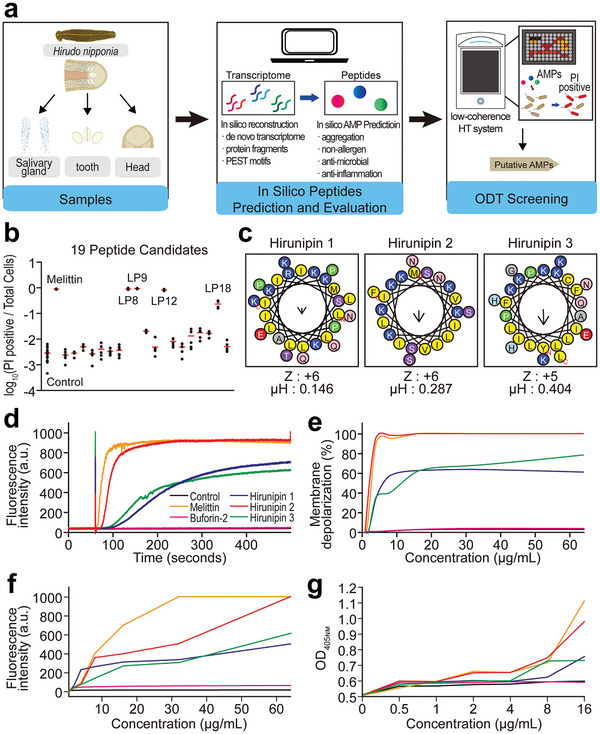
a) Experimental design and workflow of AMP prediction using in silico tools and ODT‐HTS. b) Proportion of PI‐positive to total bacterial cells on 19 AMPs identified by the ODT‐HTS method. The red bar represents the mean values ± SD of each sample from three independent experiments. c) Helical wheel diagrams of hirunipin peptides determined by HeliQuest server (https://heliquest.ipmc.cnrs.fr/cgi‐bin/ComputParams.py). Positively charged residues are presented in blue circles, the negatively charged residues are in red, the hydrophilic residues are in purple, the amide residues in pink, the hydrophobic residues in yellow, and the small residues are in grey. The N‐ and C‐terminal regions of the peptide are marked by red N and C, respectively. Z: net charge, µH: hydrophobic moment. d) Cytoplasmic membrane potential variation of *S. aureus* KCTC 1621 treated with hirunipin peptides (16 µg mL^−1^). e) Concentration‐dependent depolarization induced by hirunipin peptides. f) Membrane uptake of NPN by *E. coli* (KCTC 1682) in the presence of different concentrations of hirunipin peptides. g) Hydrolysis of *ortho*‐nitrophenyl‐β‐D‐galactoside (ONPG) due to the release of cytoplasmic β‐galactosidase of *E. coli* ML‐35 cells treated with hirunipin peptides at different concentrations measured spectroscopically at 405 nm as a function of time.

To identify the putative AMPs derived from *Hirudo nipponia*, we sequentially performed in silico peptide prediction and in vitro validation, including peptide screening, using ODT‐HTS (**Figure** **1**a). Initially, 44.96 GB of Illumina short reads from three tissues (head, salivary gland, and teeth) (Table S2, Supporting Information) of *Hirudo nipponia* were assembled into 69497 transcripts, resulting in 111 Mb (**Table** **1**). Of these, 29203 (42.02%) transcripts were annotated with functional terms, and the majority of the transcripts were not mapped using the Uniport databases. Most transcripts were similar to those in the human proteome (Figure S2, Supporting Information). Additionally, the transcripts showed 95.18% completeness in the Metazoan BUSCO dataset^[^ ^23^ ^]^ (Figure S3, Supporting Information).

**Table 1:** Summaries of *de novo* transcriptome assembly and annotations.

*De novo* transcriptome assembly	
# Transcripts	69 497
Total length (bp)	111 635 342
N50	2838
Avg. flength	160 633
Max. length	50 191
Min. length	70
GC%	40.27
Translated proteins	206 080
SwissProt	29 203 (42.02%)
KEGG	25 850 (37.20%)
Eggong	179 (0.26%)
Pfam	24 635 (35.45%)
GO	28 693 (41.29%)
GO BP	25 576 (36.80%)
GO CC	26 982 (38.82%)
GO MF	25 233 (36.31%)
No hit	40 294 (57.98%)

### 2.2 AMP Prediction from Transcriptome

The assembled transcripts were translated into 206080 proteins and the proteome was converted to 159321 small peptides. As explained in the AMP prediction method section, small peptides with observed similarity at the given cutoff values were considered known AMPs, while others were considered novel AMPs, resulting in 1453 and 477 peptides, respectively (**Table** **2**). Among these, 259 and 97 peptides from known and novel AMPs, respectively, were anti‐inflammatory. Finally, we randomly selected 19 peptides for experimental screening. Thus, the features included in the AMP prediction methodology encompassed the physicochemical and structural properties of the peptides, and a familiar web server for AMP prediction was utilized. The AMP properties of the peptides were systematically assessed and used for experimental validation.

**Table 2:** Summaries of antimicrobial peptides features along with selection cut‐offs. AIP: Anti‐inflammatory peptides.

#Propensity	Methods	Descriptions/Parameters	Cutoff/Filters	# of Sequences
	Raw Sequence	Total Given Proteins Sequences		206 080
	Raw Sequence	Small Proteins/Peptides	<= 100	159 321
Molecular	EPESTFIND	Proteins contains potential protease clevage site	!=0	13 578
	AMPA	Antimicrobial Spots	!=0	33 861
	Pepstats	Total Peptides	<= 100	33 861
	Pepstats	Peptide Length	<= 50	21 252
	Pepstats	Small Proteins which have AMP peptides	<= 50	20 012
	Pepstats	Charge	>0.00	30 051
	Pepstats	Isoelectric Point (pI)	8.00 <= pI <= 12.00	25 912
Aggregation (In vivo)	Tango	AGG	<= 500.00	24 123
	Tango	Helix	0.00<= Helix <= 25.00	23 744
	Tango	Beta	25.00<= Beta <= 100.00	11 069
Aggregation (In vitro)	Aggrescan	Na4vSS	−40.00<= Na4vSS <= 60.00	31 177
Allergen	Allerdictor	Predictions	Non‐Allergen	33 718
Homologus	Blast	Novel	No Blast Hits	33 384
	Blast	Known	Blast Hits	477
CAMP	AMP	Support Vector Machine (SVM) classifier	AMP	8616
		Random Forest Classifier	AMP	9704
		Artificial Neural Network (ANN) classifier	AMP	11 744
		Discriminant Analysis classifier	AMP	9144
		Predicted >=3 out of 4 classifier	>=3	7764
Final Result Novel: 1,453 (AIP: 259), Known: 477 (AIP: 97)				

### 2.3 Antimicrobial Activity of the 19 Putative AMPs

We tested the antibacterial activity of 19 putative AMPs (**Table** **3**) against two gram‐negative (*Escherichia coli* and *Pseudomonas aeruginosa*) and two gram‐positive (*Staphylococcus aureus* and *Staphylococcus epidermidis*) standard bacterial strains. Melittin, a well‐known bee venom AMP, was used as the positive control. The Minimum inhibitory concentrations (MICs) of putative 19 AMPs were measured using the broth microdilution method. Of these 19 peptides, only three, LP8, LP9, and LP12, were found to have promising antibacterial activity, with MIC values between 32 and 128 µg mL^−1^ (**Table** **4**). We named LP8, LP9, and LP12 as hirunipin 1, 2, and 3, respectively. In particular, hirunipin 2 showed the highest antibacterial activity with MIC values comparable to those of melittin (Table 4).

**Table 3:** Final list of the candidate antimicrobial peptides identified in the medicinal leech genome assembly by the computational algorithms.

Peptides	Amino acid sequence	Length, a.a.	Molecular weight [Da]
LP1	KLTPKPAKKVKKVVKF	16	1839.47
LP2	FGQKVNCILNFICYKYLRI LV	21	2677.40
LP3	RRRTCKKEDGTCLGRKVQYKKYLQKK	26	3214.98
LP4	KDVTGYWVRERQCKLNSKPCVGRNTQRRKYLNK	33	4025.84
LP5	LLAHVSKGFKLSMQKSKKPSLPIKT	25	2767.56
LP6	LSKGGFKGKPERAMAHHLKNIRKKLSKSIMKS	32	3607.57
LP7	IIIIKMHKTRKEKTQSILSAPVGLLTTL	28	3134.06
LP8 (Hirunipin 1)	LLKKLLRSLIKPAIMKIINTPPQEKLQK	28	3256.32
LP9 (Hirunipin 2)	MKSINLKKVVKSIIKKILNSSF	22	2519.31
LP10	KKLLKKLEKISKKKKKDKKRGRGSPMTSYAILYDGA	36	4137.24
LP11	VLNVQSIMSWNKRVGGMILNLKT	23	2602.31
LP12 (Hirunipin 3)	YFKIIKFLPKALKCLLPQKHKEHGNL	26	3108.01
LP13	NITDYNKKKVRLLPRFIFPLG	21	2533.18
LP14	LNTPRPELSISQRLLIKNFILII	23	2692.44
LP15	ARSIKKVIFSSSSRVCPPLKMLKLRPCFKKVPKLDGQ	37	4187.37
LP16	FRSQFPICKNLVNHTKFVCQKKPLSKLMKCKKALDR	36	4279.45
LP17	PKKVRKSFKKLRNFSNKNSNIYGLPA	26	3035.72
LP18	MANPVNSIKRIIKKLKKERKCKPNL	25	2950.83
LP19	QKFNGKSYALAKKMKKRQVKFLNRILDQL	29	3495.41

**Table 4:** Antimicrobial activity of the candidate antimicrobial peptides.

Peptides	Minimum inhibitory concentration (MIC^a^): µg mL^−1^			
	Gram‐negative bacteria		Gram‐positive bacteria	
	*Escherichia coli* [KCTC 1682]	*Pseudomonas aeruginosa* [KCTC 1637]	*Staphylococcus aureus* [KCTC1621]	*Staphylococcus epidermidis* [KCTC 1917]
LP1	>128	>128	>128	>128
LP2	>128	>128	>128	>128
LP3	>128	>128	>128	>128
LP4	>128	>128	>128	>128
LP5	>128	>128	>128	>128
LP6	>128	>128	>128	>128
LP7	>128	>128	>128	>128
LP8 (Hirunipin 1)	64	32	64	32
LP9 (Hirunipin 2)	32	32	32	32
LP10	>128	>128	>128	>128
LP11	>128	>128	>128	>128
LP12 (Hirunipin 3)	128	128	128	64
LP13	>128	>128	>128	>128
LP14	>128	>128	>128	>128
LP15	>128	>128	>128	>128
LP16	>128	>128	>128	>128
LP17	>128	>128	>128	>128
LP18	>128	>128	>128	>128
LP19	>128	>128	>128	>128
Melittin	32	32	16	32

Furthermore, we validated the antimicrobial effects of hirunipins using a novel peptide screening method based on ODT technology. We employed the ODT‐HTS to simultaneously monitor multiple samples of the 19 AMPs. Hirunipins 1, 2, and 3 led to a significant reduction in bacterial cells and a high proportion of PI‐positive dead cells (Figure 1b). These results suggest that hirunipins 1, 2, and 3 are highly effective AMPs, exhibiting effective antimicrobial effects compared to the other peptides. Consequently, we identified three AMPs, hirunipin 1, 2, and 3, from *Hirudo nipponia* using a prediction framework and verified them using a new approach, ODT‐HTS.

### 2.4 Molecular Structures

First, we predicted the tertiary structures of hirunipins (hirunipins 1, 2, and 3) using the I‐TASSER server. Hirunipins 1 and 3 exhibited long α‐helices at the N‐terminal region, whereas hirunipin 2 had a long α‐helix at the middle region (Figure S4, Supporting Information). We also constructed the α‐helical wheel diagrams (Figure 1c) of the peptides using the HeliQuest server. As shown in Figure 1c, the hydrophobic residues (L, I, and Y) of the peptides were clustered on one face of the helix, and the hydrophilic residues (K and R) were on the opposite face, forming amphipathic structures that are characteristic of natural AMPs. As depicted in their α‐helical wheel diagrams (Figure 1c), the hydrophilic and hydrophobic regions are less pronounced in hirunipin 1 compared to the other two peptides. This suggests that the amphipathicity of hirunipin 1 may be lower than that of hirunipins 2 and 3. This is consistent with the value of hydrophobic moment (µH), a quantitative indicator of amphipathicity. To validate the predicted structure, we measured the secondary structure of hirunipin peptides in aqueous buffer, 50% TFE, and 30 mm SDS using circular dichroism (CD) spectroscopy (Figure S5, Supporting Information). CD spectra showed that the hirunipin peptides adopted a random coil conformation in aqueous buffer, with a negative peak at 200 nm. In contrast, in 50% TFE and 30 mm SDS, which resembles the membrane environment, hirunipin peptides adopted a typical α‐helical conformation with two negative peaks ≈208 and 222 nm. Hirunipin 2 and 3 exhibited much higher α‐helical content than hirunipin 1 (Table S3, Supporting Information).

### 2.5 Hydrophobicity

We performed reversed‐phase high‐performance liquid chromatography (RP‐HPLC) to examine the antimicrobial activity of the three hirunipins. Our results showed that the retention time (hydrophobicity) of the three hirunipins was in the order hirunipin 1 (20.89 min) < hirunipin 3 (21.56 min) < hirunipin 2 (27.25 min), suggesting that hirunipin 2 exhibited the highest level of hydrophobicity (Figure S6, Supporting Information).

### 2.6 Hemolytic Activity

The hemolytic activity of hirunipins was examined on sheep erythrocytes at concentrations from 1 to 128 µg mL^−1^. Hirunipin peptides exhibit remarkably low hemolytic activity. At the highest tested concentration of 128 µg mL^−1^, these peptides caused lysis in less than 10% of erythrocytes (Figure S7, Supporting Information).

### 2.7 In Vitro Cytotoxicity

The primary limitation of AMPs in clinical applications is their potential toxicity.^[^ ^24^ ^]^ Therefore, we assessed the cytotoxicity of hirunipin peptides using the MTT assay on RAW264.7 (mouse macrophage), NIH‐3T3 (mouse fibroblast), and HaCat (human skin keratinocyte) cells. As shown in Figure S8 (Supporting Information), the cell viabilities of RAW264.7, NIH‐3T3, and HaCat cells were > 70% when exposed to 32 µg mL^−1^ of hirunipin peptides. The IC_50_ (the concentration causing 50% cell death) values of hirunipin peptides for RAW264.7, NIH‐3T3, and HaCat cells were 45–60, 50.6–57.6, and 42–64> µg mL^−1^, respectively (Table S4, Supporting Information). These results suggest that hirunipin peptides have relatively low cytotoxicity in mammalian cells, indicating their potential for therapeutic use.

### 2.8 Salt and Serum Resistance

The antimicrobial activity of AMPs can be inhibited by elevated salt concentrations;^[^ ^25^ ^]^ therefore, we evaluated the salt resistance of hirunipin peptides by measuring their MICs against *E. coli* (KCTC 1682) and *S. aureus* (KCTC 1621) in the presence of physiological levels of mono‐, di‐, and tri‐valent salts (**Table** **5**). We found that hirunipins 1 and 3 were less effective in killing bacteria under high‐salt conditions. In contrast, hirunipin 2 maintained or enhanced its antimicrobial activity in the presence of salt ions. Furthermore, to assess the serum stability of the hirunipin peptides, we measured their activity against *E. coli* and *S. aureus* in 25% human serum and observed only a 2‐ to 4‐fold decrease in activity (Table 5), suggesting resistance to serum protease degradation.

**Table 5:** MIC values of hirunipin peptides in the presence of physiological salts and human serum against *E. coli* and *S. aureus*.

Peptides	Control	150 mm NaCl	4.5 mm KCl	6 µm NH_4_Cl	1 mm MgCl_2_	2.5 mm CaCl_2_	4 µm FeCl_3_	25% Human serum
	MIC [µg mL^−1^] against *E. coli* (KCTC 1682)							
Hirunipin 1	64	256	256	128	256	256	128	128
Hirunipin 2	32	16	8	8	32	8	8	64
Hirunipin 3	128	128	128	64	256	32	32	256
	MIC [µg mL^−1^] against *S. aureus* (KCTC1621)							
Hirunipin 1	64	256	256	256	256	256	64	256
Hirunipin 2	32	32	32	16	32	32	32	64
Hirunipin 3	128	256	256	256	256	256	>256	>256

### 2.9 Antibacterial Activity Against Clinically Isolated MDR Bacteria

We evaluated the antibacterial activity of hirunipin peptides against clinically isolated MDR gram‐negative pathogens such as *P. aeruginosa*, *A. baumannii*, *K. pneumoniae*, *E. cloacae*, and *E. coli*. As shown in **Table** **6**, hirunipin 2 showed much higher antibacterial activity than hirunipins 1 and 3 and was comparable to that of melittin against MDRAP, MDRAB, and MDRPA. In particular, hirunipin 2 showed twice the antibacterial activity of melittin against MDRE and MDREC cells.

**Table 6:** Antimicrobial activity of hirunipin peptides against clinically isolated multidrug‐resistant bacteria.

Bacterial strains	Minimum inhibitory concentrations [MIC: µg mL^−1^]			
	Hirunipin 1	Hirunipin 2	Hirunipin 3	Melittin
MDRKP (328‐83)	>128	128	>128	128
MDRAB (329‐53)	>128	64	>128	32
MDRPA (314‐69)	64	16	>128	32
MDRE (328‐84)	>128	32	>128	64
MDREC (329‐66)	64	16	128	32

Clinically isolated multidrug‐resistant bacteria were obtained from Hospital of Chosun University, Korea;

MDRKP: multidrug‐resistant *Klebsiella pneumonia;*

MDRAB: multidrug‐resistant *Acinetobacter baumannii;*

MDRPA: multidrug‐resistant *Pseudomonas aeruginos*a;

MDRE: multidrug‐resistant *Enterobacter cloacae;*

MDREC: multidrug‐resistant *Escherichia coli*.

### 2.10 Antibacterial Mechanism

To gain insight into the antibacterial mechanisms of hirunipin peptides, we performed membrane depolarization, outer and inner membrane permeabilization assays, *ortho*‐nitrophenyl‐β‐D‐galactoside (ONPG) assay, propidium iodide (PI)‐influx assay using flow cytometry, scanning electron microscopy (SEM), and ODT. First, the effect of hirunipin peptides on membrane integrity was assessed, and then we measured the membrane potential of *S. aureus* (KCTC 1621) using the membrane potential‐sensitive dye 3,3′‐dipropylthiadicarbocyanine iodide (diSC_3_‐5).^[^ ^26^, ^27^ ^]^ This dye accumulates in the plasma membrane and quenches its own FL when mixed with *S. aureus* cells. However, if the membrane is depolarized by peptides or 0.1% Triton, the dye is released and its FL increases. Buforin‐2 and melittin were used as controls for intracellular and membrane‐targeting AMPs, respectively. We monitored the FL changes for 500 s after adding hirunipin peptides at 16 µg mL^−1^ (Figure 1d). As expected, buforin‐2 did not affect membrane potential. In contrast, similar to melittin, hirunipin peptides caused significant membrane depolarization at 16 µg mL^−1^. Hirunipin 2 was the most potent peptide, inducing almost 100% depolarization at 4–8 µg mL^−1^ (Figure 1e). Hirunipins 1 and 3 induced ≈60% depolarization at 16 µg mL^−1^ (Figure 1e). Depolarization was concentration‐dependent for all the peptides (Figure 1e). These results indicated that hirunipin 2 had the strongest membrane‐disrupting activity among the three peptides, which is consistent with its antibacterial ability.

Similarly, 1‐N‐phenylnaphthylamine (NPN) was used to investigate the effects of AMPs on the permeability of the bacterial outer membrane. The FL dye NPN can access the normally hydrophobic environment of the membrane when disrupted by AMPs, leading to a marked increase in FL intensity.^[^ ^26^, ^27^ ^]^ In our study, similar to melittin, hirunipin peptides caused a dose‐dependent increase in the outer membrane permeability of *E. coli* (Figure 1f). Additionally, the permeability of the bacterial inner membrane was assessed using *E. coli* ML‐35, and our results showed that hirunipin peptides, such as melittin, increased the permeability of the inner membrane in a dose‐dependent manner (Figure 1g).

**Figure 2:**
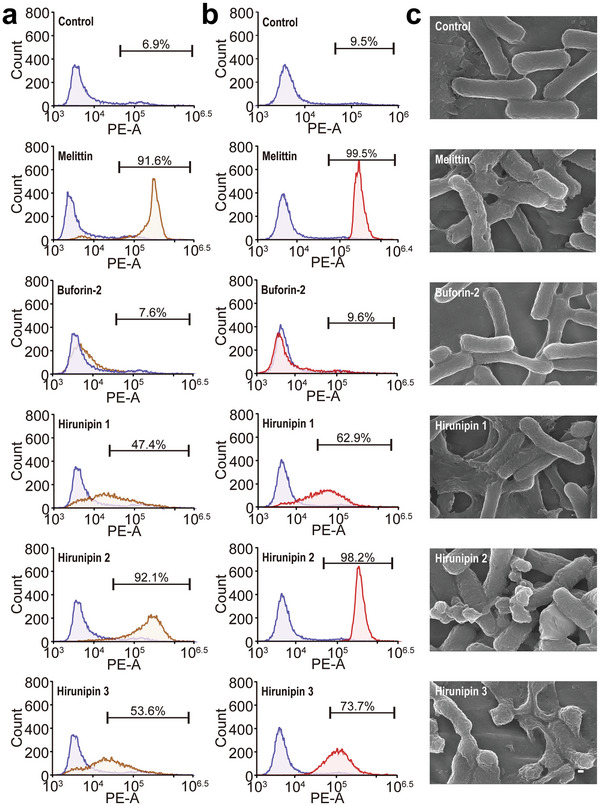
Flow cytometric analysis of *E. coli* (KCTC 1682) a) and *S. aureus* (KCTC 1621) b) treated with hirunipin peptides (16 µg mL^−1^) for 60 min and stained with PI. c) SEM images of *E. coli* (KCTC 1682) treated with the peptides (16 µg mL^−1^). Mid‐logarithmic‐phase bacteria cells were treated with hirunipin peptides at 16 µg mL^−1^ for 4 h. The control group was analyzed without treatment. Scale bar: 200 nm.

Moreover, to evaluate the potential of hirunipin peptides to permeate the bacterial cell membrane, we performed a PI influx assay using flow cytometry (**Figure** **2**a,b). Without peptide treatment, the percentage of exposed *E. coli* (KCTC1682) (Figure 2a) and *S. aureus* (KCTC 1621) cells (Figure 2b) that showed FL of PI was 6.9 and 9.5%, respectively, indicating that their membranes were intact. After treatment with 16 µg mL^−1^ of hirunipin 1, 2 and 3, the percentage of PI‐positive cells in *E. coli* increased to 47.4, 92.1, and 53.3%, respectively (Figure 2a). Similarly, the percentage of PI‐positive cells in *S. aureus* increased to 62.9, 98.2, and 73.7%, respectively (Figure 2b). These results suggest that hirunipin peptides exert antimicrobial effects against *E. coli* and *S. aureus* by disrupting their cell membranes, similar to melittin. In contrast, buforin‐2 did not cause any membrane damage, as indicated by the low percentages of PI‐positive cells in *E. coli* (KCTC1682) and *S. aureus* (KCTC 1621) (7.6% and 9.6%, respectively) (Figure 2a,b). In addition, the FL of the PI of hirunipin 2 was the highest among all the peptides (Figure 2b), which is consistent with its antibacterial activity.

Furthermore, to investigate the effects of hirunipin peptides on the morphology and surface of bacterial cells, we exposed *E. coli* (KCTC1682) to 32 µg mL^−1^ of each peptide for 4 h and analyzed them by SEM (Figure 2c). Without peptide treatment, bacterial cells exhibited normal shapes and smooth membrane surfaces (Figure 2c). Buforin‐2 did not damage bacterial surfaces. In contrast, *E. coli* cells treated with hirunipin peptides (32 µg mL^−1^) exhibited severe cell wall damage, including a rough membrane structure, holes in the cell wall, and irregularly shaped cells, similar to melittin (Figure 2c). Hirunipins 2 and 3 caused more drastic changes in the shape and structure of the bacterial cells than hirunipin 1 (Figure 2c).

**Figure 3:**
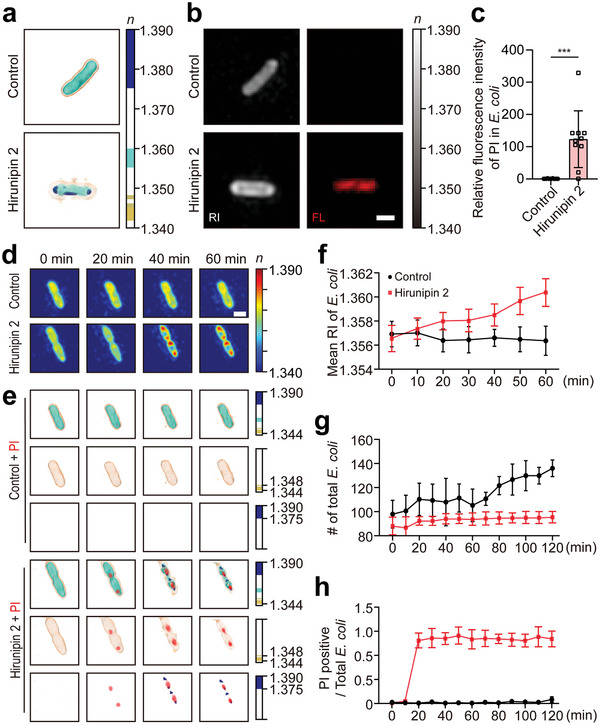
a) Representative 3D‐ODT Images of untreated (control) and hirunipin 2‐treated *E. coli* after 1 h. b) Representative 2D‐ODT and FL images for PI internalization into *E. coli*. c) Quantification analysis for FL intensity of PI in control and hirunipin 2 treated *E. coli* after 1 h. d) Time‐lapse ODT images for control and hirunipin 2 (1× MIC) treated *E. coli* for 1 h. e) Segmented ODT images including PI signal for control and hirunipin 2 treated *E. coli*. f) Quantitative analysis of mean RI of *E. coli* for control and hirunipin 2 (1× MIC) treatment for 1 h. g) Quantitative analysis of counted number of *E. coli* in FOV for control and hirunipin 2 (1× MIC) treated *E. coli* using ODT over time. h) Quantitative analysis of proportion of PI‐positive/total bacterial cells using ODT over time. Each color bar indicates the RI value. Scale bars = 1 µm. All values were presented as mean ± SD. ^*^p < 0.05, ^**^p < 0.01, and ^***^p < 0.001.

Next, we used 3D‐ODT imaging to evaluate the bactericidal effects of hirunipin 2. We obtained 3D‐ODT images of a single bacterial cell untreated or treated with hirunipin 2 to visualize the intracellular region (RI = 1.355–1.390) and cell inner membrane (RI = 1.347–1.348) and outer membrane (RI = 1.344–1.345) of *E. coli* indicated by the RI map (**Figure** **3**a). Representative ODT or FL images showed an increased mean RI of the intracellular region in hirunipin 2 treated *E. coli* compared to that in untreated cells (Figure 3b). We also observed increased PI signals in *E. coli* after treatment with hirunipin 2 using FL images and intensity quantification analysis (Figure 3b,c). Next, we tested whether the mean RI of *E. coli* increased in real‐time by analyzing the segmented 3D‐ODT images of the inner membrane and intracellular components (Figure 3d,e). Consequently, we observed that the PI intensity and mean RI of the cells gradually increased and intracellular aggregation was promoted upon treatment with hirunipin 2 (Figure 3d–f). This indicates that hirunipin 2 leads *E. coli* to membrane disruption and intracellular aggregation following cell death. Additionally, using ODT‐HTS, we confirmed a gradual decrease in the number of bulk bacterial cells, along with an increased proportion of PI‐positive cells, following treatment with hirunipin 2 over time (Figure 3g,h). Taken together, our findings suggest that hirunipin 2 exerts antibacterial effects by disrupting bacterial cell membranes and inducing intracellular aggregation, eventually leading to cell death.

### 2.11 Antibiofilm Activity

**Figure 4:**
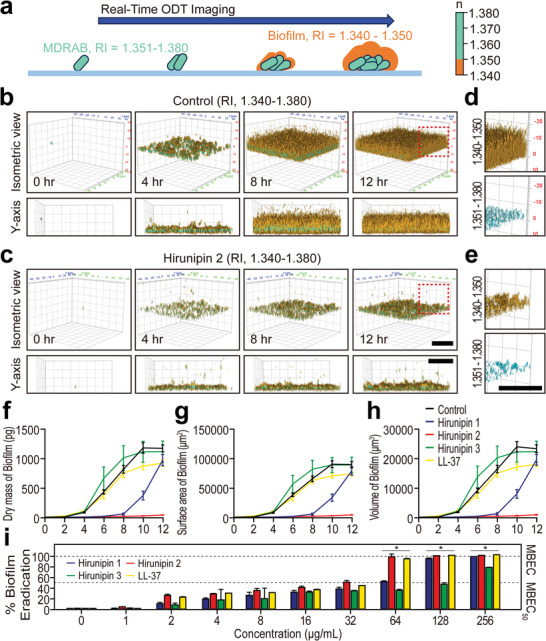
a) Experimental design of real‐time ODT imaging of biofilm formation and color map of RI. b,c) Representative 3D‐ODT images of MDRAB biofilms untreated as (b) control or treated with (c) hirunipin 2 over 12 h. Images for each time are shown in isometric view at the top and Y‐axis view at the bottom. d,e) Segmented 3D‐ODT images of MDRAB biofilm for (d) control or (e) hirunipin 2 for insets of Figure 4b,c. Images are shown in specific RI range (top, RI = 1.340‐1.350; bottom, RI = 1.351‐1.380). f–h) Quantitative analysis for (f) dry mass, (g) surface area, and (h) volume of biofilm after AMP treatment. i) Eradication activities of hirunipin peptides and LL‐37 against MDRAB (329‐53) strain. Each color bar indicates the 3D‐rendered RI distribution range (from 1.340 to 1.380). Scale bars = 30 µm. All values were presented as mean ± SD.

We visualized MDRAB biofilm formation via segmented 3D‐ODT images. We also used the RI distribution of living bacterial cells (RI = 1.351–1.380) and extracellular components of biofilms (RI = 1.340–1.350) (**Figure** **4**a). Next, we identified gradually increasing biofilm formation in the control group over time (Figure 4b; Figure S9a, Supporting Information). However, hirunipin 2 caused a severe decrease in living MDRAB and biofilm (Figure 4c) compared with LL‐37, a well‐known antibiofilm peptide (Figure S9b, Supporting Information). Additionally, hirunipin 1 (Figure S9c, Supporting Information) and hirunipin 3 (Figure S9d, Supporting Information) had less of an effect and increased the intensity of MDRAB cells. The y‐axis view enabled the observation of changes in biofilm thickness over time (Figure 4b,c; Figure S9a−d, Supporting Information). Apparently, hirunipin 2 has a severe effect of antibiofilm compared to other hirunipins and LL‐37 (Figure 4d,e; Figure S9e−h, Supporting Information). Quantitative analysis of biophysical properties, such as dry mass, surface area, and volume of the biofilm, showed that hirunipin 2 was more effective than LL‐37, hirunipin 1, or hirunipin 3 (Figure 4f–h). These results indicate that hirunipin 2 inhibits biofilm production and affects MDRAB growth. Consequently, we visualized and analyzed the reduced dynamics of biofilm after treatment with hirunipin 2 to elucidate antibiofilm mechanism applying time‐lapse label‐free ODT monitoring. Furthermore, we examined the effects of hirunipin peptides on the eradication of MDRAB biofilms using minimal biofilm eradication concentration (MBEC). Figure 4i shows that hirunipin peptides eliminated preformed MDRAB biofilms in a concentration‐dependent manner. Hirunipins 1, 2, and 3 eradicated mature MDRMA biofilms by ≈50% at concentrations of 64, 32, and 256 µg mL^−1^, respectively (Figure 4i). Therefore, the antibiofilm activity of the hirunipin peptides decreased in the order hirunipin 3 < hirunipin 1 < hirunipin 2.

### 2.12 Tendency to Develop Antimicrobial Resistance

We evaluated the drug resistance of hirunipin 2 toward *E. coli* (KCTC1682) by a multipassage resistance study. Hirunipin 2, ciprofloxacin or tetracycline was continuously exposed to *E. coli* (KCTC1682) at sub‐inhibitory concentrations. As shown in Figure S10 (Supporting Information), after 15 passages, the fold‐change in the MICs of hirunipin 2 against *E. coli* (KCTC1682) remained unchanged and stable, suggesting that hirunipin 2 can effectively prevent the development of bacterial resistance. In contrast, the fold change in the MICs of tetracycline and ciprofloxacin increased by 32‐ and 4096‐fold, respectively, indicating that the development of bacterial resistance induced by tetracycline and ciprofloxacin was significant (Figure S10, Supporting Information).

### 2.13 Synergistic Antimicrobial and Antibiofilm Activity of Hirunipin 2 with Conventional Antibiotics Against MDRAB

The synergistic effects of hirunipin 2 and conventional antibiotics against MDRAB were investigated using a checkerboard assay (**Table** **7**). For this purpose, we selected conventional antibiotics that act on different targets, such as protein (chloramphenicol and tetracycline) and DNA synthesis (ciprofloxacin and rifampicin). The fractional inhibitory concentration index (FICI) indicates the bactericidal efficiency of a drug combination; a lower FICI value indicates a better synergistic effect. The FICI values for the combination of hirunipin 2 and the four conventional antibiotics against MDRAB are summarized in Table 7. Hirunipin 2 showed very effective synergy with all antibiotics tested against MDRAB. Particularly, hirunipin 2 displayed the best synergy with chloramphenicol, with a FICI of 0.1875.

**Table 7:** FICI for the synergistic effect of hirunipin 2 in combination with antibiotics against MDRAB.

Antibiotics	MIC_A_	[A]	FIC_A_	MIC_B_	[B]	FIC_B_	FICI	Interpretation
CHL	1024	128	0.125	64	4	0.0625	0.1875	synergy
CIP	128	32	0.25	64	4	0.0625	0.3125	synergy
TET	16	2	0.125	64	8	0.125	0.25	synergy
RIF	16	1	0.0625	64	16	0.25	0.3125	synergy

CHL: Chloramphenicol, CIP: Ciprofloxacin, TET: Tetracycline, RIF: Rifampicin;

MIC: Minimal inhibitory concentration;

MIC_A_: MIC (µg mL^−1^) of antibiotic alone; [A]: MIC (µg mL^−1^) of antibiotic in combination;

MIC_B_: MIC (µg mL^−1^) of hirunipin 2 alone; [B]: MIC (µg mL^−1^) of hirunipin 2 in combination;

FIC_A_: fractional inhibitory concentration of antibiotic ([A] / MIC_A_);

FIC_B_: fractional inhibitory concentration of hirunipin 2 ([B] / MIC_B_);

FICI: fractional inhibitory concentration index, FICI = FIC_A_ + FIC_B_;

FICI ≤ 0.5 was interpreted as synergy; 0.5 < FICI ≤ 1.0 as additive; 1.0 < FICI ≤ 4.0 as indifferent; and FICI > 4.0 as antagonism.

**Figure 5:**
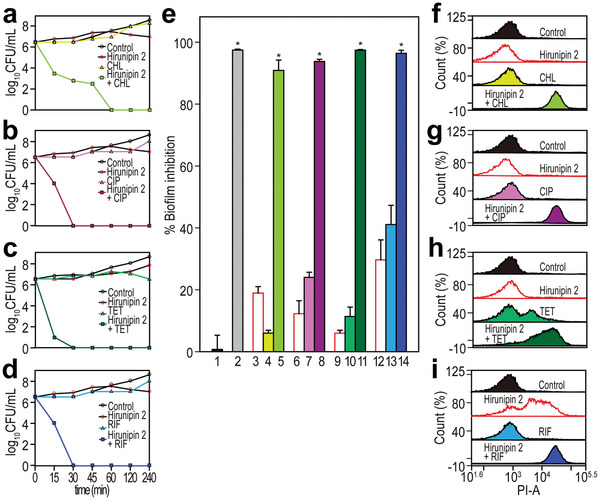
a–d) Time‐killing curve of hirunipin 2 and antibiotic alone or in combination at synergistic concentrations against MDRAB (329‐53) strain. Y‐axis indicates CFU in log scale. The values are expressed as the mean ± SEM of three independent experiments and are statistically significant at ^*^ *p* < 0.05. e) Inhibitory activity on biofilm formation of hirunipin 2 combined with antibiotic against MDRAB. 1: Control, 2: 0.1% Triton‐X100, 3: hirunipin 2 (4 µg mL^−1^), 4: CHL (128 µg mL^−1^), 5: hirunipin 2 (4 µg mL^−1^) + CHL (128 µg mL^−1^), 6: hirunipin 2 (4 µg mL^−1^), 7: CIP (32 µg mL^−1^), 8: hirunipin 2 (4 µg mL^−1^) + CIP (32 µg mL^−1^), 9: hirunipin 2 (8 µg mL^−1^), 10: TET (2 µg mL^−1^), 11: hirunipin 2 (8 µg mL^−1^) + TET (2 µg mL^−1^), 12: hirunipin 2 (8 µg mL^−1^), 13: RIF (1 µg mL^−1^), 14: hirunipin 2 (8 µg mL^−1^) + RIF (1 µg mL^−1^). The values are expressed as the mean ± SEM of three independent experiments and are statistically significant at ^*^ *p* < 0.05. f–i) FL intensity of PI in MDRAB (329‐53) strain after treatment with hirunipin 2, antibiotic, and hirunipin 2 and antibiotic measured through flow cytometry. f) Hirunipin 2 (4 µg mL^−1^), CHL (128 µg mL^−1^), and hirunipin 2 (4 µg mL^−1^) + CHL (128 µg mL^−1^) g) hirunipin 2 (4 µg mL^−1^), CIP (32 µg mL^−1^), and hirunipin 2 (4 µg mL^−1^) + CIP (32 µg mL^−1^) h) hirunipin 2 (8 µg mL^−1^), TET (2 µg mL^−1^), and hirunipin 2 (8 µg mL^−1^) + TET (2 µg mL^−1^) i) hirunipin 2 (16 µg mL^−1^), RIF (1 µg mL^−1^), and hirunipin 2 (16 µg mL^−1^) + RIF (1 µg mL^−1^).

Furthermore, to study the effect of the combination between hirunipin 2 and antibiotics on the growth kinetics of MDRAB, a time‐killing assay of hirunipin 2 alone or in combination with antibiotics, on the growth kinetics of MDRAB was conducted at concentrations that showed synergistic effects (FICI < 0.5) in the checkerboard assay. As shown in **Figure** **5**a–d, monotherapy with hirunipin 2 or antibiotics at their synergistic concentrations did not result in any bacterial killing for 4 h. However, the combination of hirunipin 2/antibiotic at their synergistic concentrations showed good synergistic and bactericidal activity within 30–60 min (Figure 5a–d). In summary, the combination of antibiotics and hirunipin 2 enhanced the cytotoxic activity against drug exposure.

Subsequently, we examined the effect of hirunipin 2, in combination with conventional antibiotics, on the inhibition of MDRAB biofilm formation. We used crystal violet staining to measure biofilm biomass. Our results showed that neither hirunipin 2 nor the antibiotics alone significantly inhibited biofilm formation at their synergistic concentrations (Figure 5e). However, the combination of hirunipin 2/chloramphenicol, hirunipin 2/ciprofloxacin, hirunipin 2/tetracycline, and hirunipin 2/rifampicin at their synergistic concentrations showed potent synergistic activity in inhibiting MDRAB biofilm formation (Figure 5e).

### 2.14 Mechanism of Synergistic Effect of Hirunipin 2 with Antibiotics Against MDRAB

To elucidate the mechanism underlying the synergistic effects of hirunipin 2 and antibiotics against MDRAB, we investigated the cell membrane permeability of MDRAB using flow cytometry (Figure 5f−i). Flow cytometric analysis revealed that incubation of the cells with the MIC of each antibiotic did not affect cell membrane permeability (Figure 5f−i). However, incubation of the cells with the MIC (64 µg mL^−1^) of hirunipin 2 resulted in a significant enhancement in FL intensity owing to PI uptake and DNA binding, indicating that the integrity of the cell membrane gradually decreased (Figure 5f−i). The hirunipin 2‐alone treatment at its synergistic concentrations (4, 8, and 16 µg mL^−1^) did not cause complete membrane permeability. However, when MDRAB was co‐treated with 4 µg mL^−1^ hirunipin 2 and 128 µg mL^−1^ chloramphenicol, corresponding to their synergistic concentrations, complete membrane damage was observed (Figure 5f). Co‐treatment with synergistic concentrations of hirunipin 2/ciprofloxacin, hirunipin 2/tetracycline, and hirunipin 2/rifampicin also induced complete membrane permeability (Figure 5g−i). These results suggest that hirunipin 2 enhances the bactericidal activity of antibiotics by increasing outer membrane permeability.

### 2.15 Anti‐inflammatory Activity in LPS‐Stimulated Murine Macrophage RAW 264.7 Cells

We also investigated the effects of hirunipin 2 on the production of proinflammatory cytokines, including TNF‐α, IL‐6, and MCP‐1 in lipopolysaccharide (LPS)‐stimulated RAW264.7 cells. Our results showed that 16 µg mL^−1^ of hirunipin 2 significantly inhibited the production of TNF‐α, IL‐6, and MCP‐1 in LPS‐stimulated RAW 264.7 by 40%, 70%, and 90%, respectively (Figure S11a−c, Supporting Information). Additionally, hirunipin 2 substantially suppressed the mRNA expression of these cytokines in LPS‐stimulated RAW264.7 cells (Figure S11d, Supporting Information), indicating its anti‐inflammatory properties.

## 3 Discussion

In this study, we identified potential AMPs in the leech genome (*Hirudo nipponia*) using a novel peptide screening strategy based on ODT technology. Unlike other label‐free methods such as confocal reflectance microscopy, raman spectroscopy, ODT offers distinct advantages and unique features. It produces 3D volumetric images with quantitative measurements of cellular properties, making it ideal for studying dynamic processes in live cells. By measuring intrinsic physical properties like RI without staining, ODT provides unique insights into cell morphology, biofilm thickness, and density in real time. Additionally, it enables fast, real‐time imaging of dynamic changes, such as AMP‐induced morphological alterations and biofilm disruption. Thus far, ODT imaging techniques have been utilized to track individual bacterial cells in real time using PI uptake and RI changes to identify antimicrobial mechanisms such as membrane disruption caused by AMPs.^[^ ^22^ ^]^ While single‐cell imaging allows for the detailed observation of individual cells, it lacks the ability to capture the collective dynamics and interactions within a larger bacterial community. To overcome this issue, we attempted time‐lapse monitoring using an ODT with a wider field‐of‐view (FOV) to simultaneously capture the bulk of bacterial cells. Using a recently developed AI‐based RI analysis, we calculated and segmented the RI values in multiple bacterial cells.^[^ ^28^ ^]^ This enabled us to distinguish numerous bacterial cells and monitor their changes over time, such as doubling time or cell death. Furthermore, we applied ODT‐HTS to verify the newly discovered 19 AMP candidates using in silico AMP prediction. Specifically, we showed that hirunipins 1, 2, and 3, which were selected by in silico and ODT‐HTS assays, were effective AMPs by cross‐validation to exhibit reliable AMP effects using conventional in vitro assays (Figures 1 and 2). The ODT‐HTS platform could also potentially be used to study various antibacterial mechanisms (Figure 3). In the future, we will provide new insights into the antimicrobial mechanisms, including changes in pH and ROS levels.^[^ ^29^ ^]^

3D imaging and quantitative analysis were performed to evaluate the antibiofilm efficacy of AMP against MDR bacteria using confocal laser scanning microscopy (CLSM)^[^ ^30^ ^]^ and Raman spectroscopy.^[^ ^31^ ^]^ Additionally, there have been a few attempts to image and quantitatively analyze the dynamics of bacterial biofilm formation in vitro using digital holographic tomography.^[^ ^32^, ^33^ ^]^ Although label‐free imaging has been used to observe biofilm dynamics, it has not been applied to the HTS screening of AMP. Interestingly, our study showed that the ODT‐HTS for simultaneous time‐lapse monitoring could be utilized to perform a comparative analysis of antibiofilm mechanisms induced by AMPs. Although this enables the tracking of biofilm dynamics by culture, there are several considerations for improving the reliability of our study. First, because it is an imaging technique based on RI changes induced by ODT, we must develop a biofilm marker to verify which components are present in the biofilm. Currently, anti‐DNA or WGA staining can be used to visualize biofilms by marking extracellular DNA or polysaccharide elements in the biofilm.^[^ ^34^, ^35^ ^]^ Second, there are limitations in observing biofilm dynamics using ODT. The biofilm becomes thicker over time, and excessive biofilm production blocks light transmission and interferes with long‐term ODT imaging. Moreover, it is difficult to distinguish the components within a biofilm, especially planktonic cells, during time‐lapse monitoring. Lastly, challenges related to data volume and resolution still remain limitations in the application of ODT. To mitigate these challenges, the integration of AI‐based automated processing has proven effective for managing and analyzing large datasets. Regarding resolution, the characteristics of the light source are particularly critical, as both light penetration and the amount of light information significantly influence image quality. Future advancements in ODT technology, especially in optimizing light sources, will be essential to address these issues and further enhance the system's performance and applicability. Nevertheless, we believe that these limitations can be overcome, and this approach represents a novel attempt at identifying antibiofilm efficacy through the imaging of initial biofilm formation.

The main challenge in discovering AMPs is that they have strong antibacterial activity against bacterial cells and are nontoxic to mammalian cells.^[^ ^24^ ^]^ The hirunipin peptides discovered in this study showed weak hemolytic activity of < 10% even at a high concentration of 128 µg mL^−1^ and showed relatively weak toxicity to mammalian cells. This demonstrated the usefulness of AMPs for therapeutic purposes. Additionally, stability in physiological salt environments and serum is another parameter that needs to be considered for the discovery of novel antimicrobial agents. Physiological salt environments and serum can hinder the antimicrobial activity of AMPs by interfering with their electrostatic interactions with the negatively charged components of bacterial membranes and in the presence of proteases and peptide‐binding proteins, respectively.^[^ ^36^, ^37^ ^]^ This poses a significant obstacle to therapeutic development. In this study, hirunipin 2 maintained its antimicrobial activity in the presence of physiological salts and human serum, indicating its potential as an antimicrobial agent. Furthermore, the concept of outer membrane permeability is crucial for understanding how certain compounds enhance antibiotic efficacy. Gram‐negative bacteria possess an outer membrane that acts as a barrier limiting the entry of molecules, including antibiotics.^[^ ^38^ ^]^ By increasing the membrane permeability, hirunipin 2 allows antibiotics to bypass this barrier and exert their effects more effectively (Figure 5). This combination therapy may offer new clinical options for combating antibiotic‐resistant bacterial strains.

Biofilm formation is a potential cause of many infections and is resistant to antimicrobial agents. Approximately 65% of all bacterial infections are associated with bacterial biofilms. To combat bacterial biofilms, antimicrobial agents must prevent biofilm formation and break down mature biofilms.^[^ ^39^, ^40^, ^41^ ^]^ Research indicates that some bacteria can rapidly form biofilms in medical environments, which poses a significant challenge. For instance, bacteria such as *Acinetobacter baumannii* are known to quickly develop biofilms in settings such as Intensive Care Units (ICUs) on the surfaces of urinary catheters, bronchial epithelial cells, and various other abiotic materials.^[^ ^42^, ^43^, ^44^ ^]^ Meanwhile, the mortality rate from MDRAB‐associated burn wound infections in patients is notably high.^[^ ^44^ ^]^ Therefore, we examined the antibiofilm properties of hirunipin peptides. Interestingly, our results showed that the antibiofilm activity of hirunipin 2 was nearly equivalent to that of LL‐37, a powerful antibiofilm AMP,^[^ ^45^, ^46^ ^]^ suggesting its potentiality as an antibiofilm agent for the treatment of MDRAB infections.

Antimicrobial resistance (AMR) is a serious global threat that necessitates the development of novel strategies to combat infections.^[^ ^47^, ^48^, ^49^, ^50^ ^]^ A promising approach is to combine different drugs to achieve synergistic effects, which can either increase the therapeutic efficacy or reduce side effects by allowing the use of lower doses.^[^ ^51^, ^52^, ^53^, ^54^ ^]^ Such combinations can also help overcome AMR and extend the lifespan of existing antibiotics.^[^ ^55^, ^56^, ^57^ ^]^ Furthermore, some AMPs have been shown to enhance the activity of conventional antibiotics in addition to their direct antimicrobial action.^[^ ^58^, ^59^, ^60^, ^61^ ^]^ In this study, the synergistic effect of hirunipin 2 with other antibiotics, chloramphenicol, ciprofloxacin, tetracycline, and rifampicin, in inhibiting biofilm formation by MDRAB clearly indicated that hirunipin 2‐antibiotic combinations could be a very effective strategy for combating MDR bacteria and biofilm‐associated infections. This study also highlights the notable anti‐inflammatory properties of hirunipin 2. LPS released during bacterial cell division or death is considered the primary trigger of sepsis.^[^ ^62^, ^63^ ^]^ The novel peptide hirunipin 2 demonstrated anti‐inflammatory properties by suppressing LPS‐induced proinflammatory cytokines, suggesting its therapeutic benefits in endotoxemia. Thus, hirunipin 2 would be a promising candidate for the treatment of sepsis‐associated infections and inflammation.

## 4 Conclusion

In summary, we demonstrated that the ODT‐HTS method can serve as a simultaneous analysis platform for identifying the antimicrobial or antibiofilm effects of AMPs. This method is a label‐free method capable of identifying cell death and AMP efficacy by analyzing the volume, dry mass, and mean RI of bacterial cells without requiring additional steps like staining or fixation. As ODT‐HTS can be readily adapted to identify bacterial cell dynamics, we suggest that it is a promising strategy for many applications, from AMP discovery to MDR research. Using this technology, we identified a novel AMP (hirunipin 2) from *Hirudo nipponia* that showed superior antibacterial efficacy with minimal hemolytic and cytotoxic effects and a reduced likelihood of inducing bacterial resistance. Additionally, the synergistic effects of hirunipin 2 with conventional antibiotics, along with its unique properties including drug resistance, antibiofilm activity, and anti‐inflammatory activity, highlight its potential as a novel leech‐derived AMP against antibiotic‐resistant infections. Therefore, our study proposes hirunipin 2 as a promising candidate for combating bacterial, biofilm, and MDR bacterial infections, offering significant advancements in addressing the global challenge of antibiotic resistance.

## 5 Experimental Section

### Illumina Library Preparation and Sequencing

Total RNA was isolated from frozen samples using the Qiagen RNA isolation kit, and the total mRNA was converted into library templates using the TruSeq RNA Sample Prep Kit v2. This procedure includes purification, strand synthesis, end repair, adapter ligation, and PCR enrichment. The enriched libraries were quantified and sequenced using Illumina Hi‐Seq 4000 and Illumina Novo‐Seq6000 respectively. Finally, the sequenced reads were processed using sequencing control software, and the outputs were paired‐end FASTQ‐formatted files. The complete procedure was performed by Macrogen Inc. (http://www.macrogen.com).

### Assembly and Differential Gene Expression

Complete short‐read sequences were preprocessed using BBduk v38.26^[^ ^64^ ^]^ to remove low‐quality chimeric sequences and adapter contaminants. Processed reads were subjected to de novo assembly using Trinity v2.11.0^[^ ^65^ ^]^ and mapped back to the reference with Bowtie2 v.2.3.3^[^ ^66^ ^]^ to filter out the expressed contigs. Furthermore, to reduce the isoforms and highly similar fragmented contigs, sequence clustering CD‐HIT‐EST v4.8.13^[^ ^67^ ^]^ was performed and translated to protein sequences using TransDecoder v5.5.0 (https://github.com/TransDecoder/TransDecoder/releases). The protein sequences were annotated with Trinotate v3.2.2 (http://trinotate.github.io) to obtain the functional terms such as gene ontology (GO), Kyoto encyclopedia of genes and genomes (KEGG) pathways, and protein domains. Finally, the completeness of the assembled transcripts was assessed using the BUSCO metazoa_odb10.2021‐02‐24 dataset.^[^ ^23^ ^]^

### AMP Prediction and Classification

The complete amino acid sequences prepared from the trans‐decoder were subjected to AMP prediction analysis using the proposed bioinformatics strategy. The peptide characteristics of molecular propensity (based on physicochemical properties) and aggregation propensity (in‐vitro and in‐vivo) were determined and AMP prediction was established using a predefined bioinformatics strategy with previously defined parameters.^[^ ^68^ ^]^ In addition to this previous strategy, the allergenic propensity of the peptides was also determined using Allerdictor software.^[^ ^69^ ^]^ Finally, AMPs were mapped using the CAMP database^[^ ^70^ ^]^ and classified as novel or known AMPs. To classify the predicted AMPs as novel, sequences were matched to the CAMP database by using two programs: PatMatch (no mismatch) for sequences ≤ 20 bp in length^[^ ^71^ ^]^ and BLASTP (1E‐05) for sequences ≥ 20 bp in length. The BLAST results were filtered with a similarity score ≥ 90. Sequences with observed similarity at the given cutoff values were considered known AMPs, and others were considered novel AMPs. Finally, the novel and known AMPs were manually validated for continuous stretching of amino acids to account for low‐complexity regions and assembly artifacts.

### Synthesis of the 19 Putative AMPs

The 19 putative AMPs used in the experiments were synthesized by Dandicure, Ltd. via Fmoc‐based solid‐phase peptide synthesis. The mass of the purified peptides was determined using matrix‐assisted laser desorption/ionization time‐of‐flight (MALDI‐TOF) mass spectroscopy. The mass data for all peptides are provided in Table S1 and Figure S1 (Supporting Information). The calculated and measured peptide weights were consistent, confirming the accurate synthesis of all 19 peptides.

### Minimal Inhibitory Concentrations (MICs)

The MICs of the peptides were measured by the Clinical and Laboratory Standards Institute (CLSI) broth microdilution method, as outlined in a previous study.^[^ ^26^, ^27^ ^]^

### Salt and Serum Sensitivity Assay

The salt sensitivity of the peptides was also analyzed. *E. coli* (KCTC 1682) and *S. aureus* (KCTC 1621) were incubated in the presence of different final concentrations of physiological salts (150 mm NaCl, 4.5 mm KCl, 6 µm NH_4_Cl, 8 µm ZnCl_2_, 1 mm MgCl_2_, 2 mm CaCl_2_, and 4 µm FeCl_3_) or human serum (20%). The MIC values in the physiological environment were determined as described above, and data were acquired from three independent assays performed in triplicate.

### CD Spectroscopy

A Jasco‐715 spectropolarimeter (Jasco) was used to perform CD analysis to evaluate the secondary structure of hirunipin peptides in a membrane‐mimetic environment, with a final peptide concentration of 150 µm. The CD spectra were measured at wavelengths ranging from 190 to 250 nm with a path length of 1 mm at room temperature. The percent α‐helical content was calculated using the equation, % α‐helical content = −100 (*θ*_222_ + 3000)/33 000, proposed by McLean et al.^[^ ^72^ ^]^

### RP‐HPLC

An HPLC system (SHIMAZU Model SPD‐M20 230V) with a UV detector (215 nm) and a manual injector (20 µL) was used in the experiment, wherein 5 µg of hirunipin peptides dissolved in distilled water was injected into the Vydac C_18_ column (5 mm; 4.6 mm × 250 mm). The column was equilibrated with 0.05% (v/v) TFA/water and eluted using a linear gradient of acetonitrile at a flow rate of 1.0 mL min^−1^.

### Hemolysis and Cytotoxicity Assays

Hemolytic activity of the peptides was assessed using sheep red blood cells (sRBCs), as outlined in a previous study. The cytotoxicity of hirunipin peptides toward mouse macrophage RAW264.7, mouse fibroblast NIH‐3T3, and human keratinocyte HaCaT cells was determined using the MTT method, as outlined in previous studies.^[^ ^26^, ^27^ ^]^

### Drug Resistance Study

The MICs of hirunipin 2 and antibiotics were determined, and the concentration of each sub‐MIC (0.5×MIC) of *E. coli* (KCTC 1682) was diluted to 1 × 10^6^ CFU mL^−1^ with MHB for the next passaged MIC determination. This procedure was repeated fifteen times. Ciprofloxacin and tetracycline were used as the controls.

### Membrane Depolarization Assay

The FL dye diSC_3_‐5 (3,3′‐dipropylthiadicarbocyanine iodide) was utilized to assess the depolarizing impact of peptides on the cytoplasmic membrane of *S. aureus* (KCTC 1621), following a previously established method.^[^ ^26^, ^27^ ^]^

### Outer Membrane Permeabilization Assay

The FL dye NPN (1‐N‐phenylnaphthylamine) was used to assess the outer membrane permeability of *E. coli* (KCTC 1682), as previously described.^[^ ^26^, ^27^ ^]^

### Flow Cytometry Analysis

Damage to membrane integrity induced by the peptides was evaluated by flow cytometry of *E. coli* (KCTC 1682) and *S. aureus* (KCTC 1621) cells stained with the membrane‐impermeable dye PI, as previously described.^[^ ^26^, ^27^ ^]^

### SEM Imaging

Morphological changes in the membranes of peptide‐treated *E. coli* (KCTC 1682) were visualized using SEM as described previously.^[^ ^26^, ^27^ ^]^

### 3D‐ODT Imaging and Quantitative Analysis for Single *E. coli*

The 3D‐ODT and FL images of living single bacterial cells were obtained following a previously described protocol with minor modifications using a commercial instrument (HT‐2H, Tomocube).^[^ ^22^ ^]^ It enables the acquisition of high‐resolution 3D RI tomograms (110 nm lateral, 220 nm axial) using a coherent laser (532 nm) and digital micromirror device. Also, this instrument features real‐time phase retrieval for accurate focusing and integrated FL imaging with three‐channel LED light sources for 3D reconstruction. Following that, each single bacterial cell was individually caught using ODT for 1 h at 10‐min intervals after treatment with PBS or hirunipin 2 (32 µg mL^−1^) containing PI (2 µg mL^−1^). Using commercial software (TomoStudio, Tomocube), the cells were visualized and quantified using voxels reconstructed from the RI, including FL imaging.

### 3D‐ODT Imaging and Quantitative Analysis for MDRAB Biofilm

For ODT imaging of biofilms, multidrug‐resistant *Acinetobacter baumannii* (MDRAB) (329‐53) cells were inoculated and grown in MHB medium. The bacterial cells were harvested by centrifugation, washed with PBS, and resuspended in fresh MHB media at 1.0 × 10^6^ cells mL^−1^ in a 6‐well plate with a high performance #1.5 coverslip bottom (Cellvis). AMPs were prepared at 64 µg mL^−1^ in MHB media and added to each well.

To monitor the morphological dynamics of the biofilms, ODT imaging was performed every two hours for 12 h using a low‐coherence HT system (HT‐X1, Tomocube). TomoAnalysis version 1.7.13 (Tomocube) was used to visualize 3D RI tomograms of biofilm and quantify the biophysical properties such as volume, dry mass, and surface area in the FOV which is set as 80 × 80 µm.

### Quantitative Analysis of FL Intensity

The FL intensity of the PI was analyzed using ImageJ software by applying a segmentation algorithm to mask the cell boundary and stained areas using Otsu's method and subsequently measuring the average integrated density at the cell boundary. The intensity values quantified in the cells were averaged.

### MBEC (Minimal biofilm Eradication Concentration)

The MBEC for peptides against the multidrug‐resistant *Pseudomonas aeruginosa* (MDRPA) CCARM 2095 strain was assessed using the Calgary Biofilm Device (CBD) supplied by Innovotech, as previously reported.^[^ ^26^, ^27^ ^]^

### Confocal Laser Scanning Microscopy (CLSM)

The biofilm eradication activity of hirunipin peptides against preformed biofilms of MDRAB (329‐53) strain was confirmed by CLSM with LIVE/DEAD staining (SYTO9/PI), as reported earlier.^[^ ^26^, ^27^ ^]^

### Synergy Testing by Checkerboard Assay

The synergy of hirunipin 2 in combination with conventional antibiotics (chloramphenicol, tetracycline, ciprofloxacin, and rifampicin) against MDRAB (329‐53) was investigated using a checkerboard assay, as reported earlier.^[^ ^26^, ^27^ ^]^

### Time Killing Assay

The time‐dependent bactericidal activity of hirunipin 2 alone or in combination with antibiotics against MDRAB (329‐53) was determined as described previously.^[^ ^26^, ^27^ ^]^

### Measurement of TNF‐α, IL‐6, and MCP‐1 Release from LPS‐Stimulated RAW264.7 Cells

Inhibition of TNF‐α, IL‐6, and MCP‐1 production induced by hirunipin 2 in LPS‐stimulated RAW 264.7 cells was determined using ELISA, as described previously.^[^ ^26^, ^27^ ^]^

### Reverse‐Transcription Polymerase Chain Reaction (RT‐PCR)

RT‐PCR analysis for quantification of TNF‐α, IL‐6, and MCP‐1 was performed, as described previously.^[^ ^26^, ^27^ ^]^

### Statistical Analysis

Statistical analysis was performed using Student's t‐test for comparison between groups or one‐way analysis of variance (ANOVA) with Tukey's multiple comparisons for comparisons between more than two groups. Statistical analyses were performed using SPSS ver. 22 (SPSS). *p < 0.05* was considered to indicate a statistically significant. All values are expressed as mean ± standard deviation (SD). Statistical analyses were performed using GraphPad Prism 8 or Origin 2018 program. Comparisons between groups were performed using unpaired and two‐tailed t‐tests. All values were presented as mean ± SD. ^*^ *p* < 0.05, ^**^ *p* < 0.01 and ^***^ *p* < 0.001.

Supplementary data provide detailed procedures for the following assays and analyses: MICs, hemolysis assay, cytotoxicity assay, drug resistance study, outer membrane permeabilization assay, flow cytometry analysis, CLSM, synergy testing by checkerboard assay, MBEC, time killing assay, measurement of TNF‐α, IL‐6, and MCP‐1 release from LPS‐stimulated RAW264.7 cells, and RT‐PCR.

## Conflict of Interest

The authors declare no conflict of interest.

## Author Contributions

S.D.K., J.P., N.K.R., and Y.P.A. contributed equally to this work., and each reserve the right to put their name first on their respective CVs. S.D.K. performed formal analysis and data collection. J.P. performed formal analysis, data collection, and wrote ‐ original draft. N.K.R. performed formal analysis and data collection. Y.P.A. performed data collection. G.‐H.J. performed data collection. I.‐H.P. performed data collection. B.G. performed data collection. C.W.L. performed data collection. S.Y. performed data collection. Y.S. performed data collection. S.S. performed data collection. Y.‐j.L. performed data collection. S.‐H.K. performed data collection. S.L. performed formal analysis, wrote ‐ original draft, acquired funding acquisition, and performed supervision. S.Y.S performed formal analysis, wrote ‐ original draft, acquired funding acquisition, and performed supervision. S.J.C. performed formal analysis, wrote ‐ original draft, acquired funding acquisition, and performed supervision. All authors gave final approval and agree to be accountable for all aspects of the work.

## Supporting information



Supporting Information

## Data Availability

The data that support the findings of this study are available from the corresponding author upon reasonable request.

## References

[1] WHO , Prioritization of Pathogens to Guide Discovery, Research and Development of New Antibiotics for Drug‐Resistant Bacterial Infections, Including Tuberculosis; World Health Organization, Geneva, Switzerland 2017 pp. 41–79.

[2] J. H. Calhoun , C. K. Murray , M. M. Manring , Clin. Orthop. Relat. Res. 2008, 466, 1356. 18347888 10.1007/s11999-008-0212-9 PMC2384049

[3] H. C. Yun , C. K. Murray , US Army Med. Dep. J. 2016 114. 27215877

[4] A. Penesyan , S. S. Nagy , S. Kjelleberg , M. R. Gillings , I. T. Paulsen , NPJ Biofilms Microbiomes 2019, 5, 34. 31728201 10.1038/s41522-019-0108-3 PMC6834608

[5] P. Gallagher , S. Baker , J. Infect. 2020, 81, 857. 33115656 10.1016/j.jinf.2020.10.016

[6] S. N. Dean , B. M. Bishop , M. L. van Hoek , Front. Microbiol. 2011, 2, 128. 21772832 10.3389/fmicb.2011.00128 PMC3131519

[7] L. S. Amer , B. M. Bishop , M. L. van Hoek , Biochem. Biophys. Res. Commun. 2010, 396, 246. 20399752 10.1016/j.bbrc.2010.04.073

[8] G. Rajasekaran , E. Y. Kim , S. Y. Shin , Biochim. Biophys. Acta Biomembr. 2017, 1895, 722. 10.1016/j.bbamem.2017.01.037 28161291

[9] X. Feng , K. Sambanthamoorthy , T. Palys , C. Paranavitana , Peptides 2013, 49, 131. 24071034 10.1016/j.peptides.2013.09.007

[10] R. E. Hancock , H. G. Sahl , Nat. Biotechnol. 2006, 24, 1551. 17160061 10.1038/nbt1267

[11] N. Mookherjee , M. A. Anderson , H. P. Haagsman , D. J. Davidson , Nat. Rev. Drug Discovery. 2020, 19, 311. 32107480 10.1038/s41573-019-0058-8

[12] J. Lei , L. Sun , S. Huang , C. Zhu , P. Li , J. He , V. Mackey , D. H. Coy , Q. He , Am. J. Transl. Res. 2019, 11, 3919. 31396309 PMC6684887

[13] R. Sawyer , in Leech Biology and Behaviour, II, Oxford University Press, New York, New York 1986 p. 1065.

[14] E. N. Grafskaia , K. D. Nadezhdin , I. A. Talyzina , N. F. Polina , O. V. Podgorny , E. R. Pavlova , P. V. Bashkirov , D. D. Kharlampieva , P. A. Bobrovsky , I. A. Latsis , V. A. Manuvera , V. V. Babenko , V. M. Trukhan , A. S. Arseniev , D. V. Klinov , V. N. Lazarev , Eur. J. Med. Chem. 2019, 180, 143. 31302447 10.1016/j.ejmech.2019.06.080

[15] A. Tasiemski , F. Vandenbulcke , G. Mitta , J. Lemoine , C. Lefebvre , P. E. Sautiere , M. Salzet , P. Sautie , M. Salzet , J. Biol. Chem. 2004, 279, 30973. 15102860 10.1074/jbc.M312156200

[16] M. Salzet , Curr. Med. Chem. 2005, 12, 3055. 16375700 10.2174/092986705774933470

[17] A. Tasiemski , Invertebr. Surviv. J. 2008, 5, 75.

[18] S. Jung , F. D. Sonnichsen , C. W. Hung , A. Tholey , C. Boidin‐Wichlacz , €. W. Haeusgen , C. Gelhaus , C. Desel , R. Podschun , V. Waetzig , A. Tasiemski , M. Leippe , J. Grotzinger , J. Biol. Chem. 2012, 287, 14246. 22396551 10.1074/jbc.M111.336495 PMC3340141

[19] S. Ghosh , Int. J. Pept. Res. Ther. 2020, 26, 2253.

[20] J. Huang , Y. Xu , Y. Xue , Y. Huang , X. Li , X. Chen , Y. Xu , D. Zhang , P. Zhang , J. Zhao , J. Ji , Nat. Biomed. Eng. 2023, 7, 797. 36635418 10.1038/s41551-022-00991-2

[21] H. Lee , S. Lee , I. Lee , H. Nam , Protein Sci. 2023, 32, e4529. 36461699 10.1002/pro.4529 PMC9793967

[22] M. Kim , Y. Cheon , D. Shin , J. Choi , J. E. Nielsen , M. S. Jeong , H. Y. Nam , S. H. Kim , R. Lund , H. Jenssen , A. E. Barron , Adv. Sci. 2023, 24, 2302483. 10.1002/advs.202302483 PMC10460844 37341246

[23] M. Manni , M. R. Berkeley , M. Seppey , E. M. Zdobnov , BUSCO: Assessing Genomic Data Quality and Beyond. Current Protocols. 2021, 1, e323, 10.1002/cpz1.323. 34936221

[24] R. N. I. Kishi , D. Stach‐Machado , J. de Lacorte Singulani , C. T. dos Santos , A. M. Fusco‐Almeida , E. M. Cilli , J. Freitas‐Astúa , S. C. Picchi , M. A. Machado , PLoS One 2018, 13, e0203451. 30192822 10.1371/journal.pone.0203451 PMC6128562

[25] J. Huang , D. Hao , Y. Chen , Y. Xu , J. Tan , Y. Huang , F. Li , Y. Chen , Peptides 2011, 32, 1488. 21664394 10.1016/j.peptides.2011.05.023

[26] E. Y. Kim , S. D. Kumar , J. K. Bang , C. Ajish , S. Yang , B. Ganbaatar , J. Kim , C. W. Lee , S. J. Cho , S. Y. Shin , Int. J. Antimicrob. Agents. 2023, 62, 106909. 37419291 10.1016/j.ijantimicag.2023.106909

[27] S. Dinesh Kumar , J. H. Park , H. S. Kim , C. D. Seo , C. Ajish , E. Y. Kim , H. S. Lim , S. Y. Shin , Eur. J. Med. Chem. 2022, 243, 114747. 36103802 10.1016/j.ejmech.2022.114747

[28] E. Y. Jeong , H. J. Kim , S. Lee , Y. Park , Y. M. Kim , Three‐dimensional Label‐Free Measurements of Adipocyte Differentiation and Lipid Droplet Dynamics, bioRxiv. 2024, 2024, 10.1101/2024.01.06.574492.

[29] J. Shi , C. Chen , D. Wang , Z. Wang , Y. Liu , Comm. Biol. 2022, 5, 926. 10.1038/s42003-022-03899-4 PMC9452538 36071151

[30] B. V. Areválo , M. Salinas‐Pena , I. Ponte , A. Jordan , A. Roque , E. Torrents , Antimicrobial and antibiofilm activity of human recombinant H1 histones against bacterial infections, bioRxiv. 2024, 2024. 10.1101/2024.04.03.587932. PMC11575268 39470247

[31] A. K. Locke , F. R. Zaki , S. T. Fitzgerald , K. Sudhir , G. L. Monroy , H. Choi , J. Won , A. Mahadevan‐Jansen , S. A. Boppart , Front. Cell. Infect. Microbiol. 2022, 12, 869761. 36034696 10.3389/fcimb.2022.869761 PMC9400059

[32] I. Buzalewicz , A. Kaczorowska , W. Fijałkowski , A. Pietrowska , A. K. Matczuk , H. Podbielska , A. Wieliczko , W. Witkiewicz , N. Jędruchniewicz , Int. J. Mol. Sci. 2024, 25, 2653. 38473902 10.3390/ijms25052653 PMC10932241

[33] I. Buzalewicz , A. Ulatowska‐Jarża , M. Gąsior‐Głogowska , M. Wolf‐Baca , P. Żyłka , Measurement 2023, 210, 112588.

[34] S. Sugimoto , Y. Kinjo , Commun. Biol. 2023, 6, 38. 36690667 10.1038/s42003-022-04396-4 PMC9870912

[35] A. R. Sultan , T. Hoppenbrouwers , N. A. Lemmens‐den Toom , S. V. Snijders , J. W. van Neck , A. Verbon , M. P. de Maat , W. J. van Wamel , Infect. Immun. 2019, 87, 128. 10.1128/IAI.00605-19 PMC6867843 31527127

[36] Y. X. Chen , M. T. Guarnieri , A. I. Vasil , M. L. Vasil , C. T. Mant , R. S. Hodges , Antimicrob. Agents Chem. 2007, 51, 1398. 10.1128/AAC.00925-06 PMC1855469 17158938

[37] S. Kim , S. S. Kim , B. J. Lee , Peptides 2005, 26, 2050. 15894405 10.1016/j.peptides.2005.04.007

[38] Y. Liu , Z. T. , J. Shi , R. Li , M. Upton , Z. Wang , Theranostics 2021, 11, 4910. 33754035 10.7150/thno.56205 PMC7978324

[39] G. Di Perri , G. Ferlazzo , New Microbiol. 2022, 45, 227. 36190373

[40] A. Y. Peleg , H. Seifert , D. L. Paterson , Clin. Microbiol. Rev. 2008, 21, 538. 18625687 10.1128/CMR.00058-07 PMC2493088

[41] R. Roy , M. Tiwari , G. Donelli , V. Tiwari , Virulence 2018, 9, 522. 28362216 10.1080/21505594.2017.1313372 PMC5955472

[42] L. C. S. Antunes , P. Visca , K. J. Towner , Pathog. Dis. 2014, 71, 292. 24376225 10.1111/2049-632X.12125

[43] F. Longo , C. Vuotto , G. Donelli , New Microbiol. 2014, 37, 119. 24858639

[44] D. F. Borges Duarte , A. G. Rodrigues , APMIS 2022, 130, 330. 35403751 10.1111/apm.13227

[45] H. Memariani , M. Memariani , World J. Microbiol. Biotechnol. 2023, 39, 99. 36781570 10.1007/s11274-023-03545-z

[46] K. E. Ridyard , J. Overhage , Antibiotics 2021, 10, 650. 34072318 10.3390/antibiotics10060650 PMC8227053

[47] World Health Organization . Antimicrobial Resistance: Global Report on Surveillance, 2014.

[48] K. M. G. O'Connell , J. T. Hodgkinson , H. F. Sore , M. Welch , G. P. C. Salmond , D. R. Spring , Angew. Chem., Int. Ed. 2013, 52, 10706. 10.1002/anie.201209979 24038605

[49] S. Lin , J. Liu , H. Li , Y. Liu , Y. Chen , J. Luo , S. Liu , J. Med. Chem. 2020, 63, 9284. 32787074 10.1021/acs.jmedchem.0c00433

[50] D. J. Farrell , M. Robbins , W. Rhys‐Williams , W. G. Love , Antimicrob. Agents. Chemother. 2011, 55, 1177. 21149626 10.1128/AAC.01285-10 PMC3067113

[51] C. Chatupheeraphat , J. Peamchai , S. Luk‐In , W. Eiamphungporn , Front. Cell Infect Microbiol. 2023, 13, 1153868. 37113135 10.3389/fcimb.2023.1153868 PMC10126264

[52] M. A. Paduszynska , K. E. Greber , W. Paduszynski , W. Sawicki , W. Kamysz , Antibiotics 2020, 9, 566. 32887236 10.3390/antibiotics9090566 PMC7560174

[53] A. Almaaytah , A. Abualhaijaa , O. Alqudah , Infect Drug Resist 2019, 12, 1371. 31213855 10.2147/IDR.S204626 PMC6537036

[54] J. Li , P. Fernández‐Millán , E. Boix , Curr. Top. Med. Chem. 2020, 20, 1238. 32124698 10.2174/1568026620666200303122626

[55] A. R. M. Coates , Y. Hu , J. Holt , P. Yeh , Expert Rev. Anti‐Infect. Ther. 2020, 18, 5. 31847614 10.1080/14787210.2020.1705155

[56] N. Wang , J. Luo , F. Deng , Y. Huang , H. Zhou , Front. Pharmacol. 2022, 13, 839808. 35281905 10.3389/fphar.2022.839808 PMC8905495

[57] G. Rajasekaran , S. D. Kumar , S. Yang , S. Y. Shin , Eur. J. Med. Chem. 2019, 182, 111623. 31473417 10.1016/j.ejmech.2019.111623

[58] E. Y. Kim , G. Rajasekaran , S. Y. Shin , Eur. J. Med. Chem. 2017, 136, 428. 28525841 10.1016/j.ejmech.2017.05.028

[59] P. R. Lennard , P. S. Hiemstra , P. S. Nibbering , Antibiotics 2023, 12, 1518. 37887219 10.3390/antibiotics12101518 PMC10604037

[60] J. T. Mhlongo , A. Y. Waddad , F. Albericio , B. G. de la Torre , Adv. Sci. 2023, 10, e2300472. 10.1002/advs.202300472 PMC10502873 37407512

[61] J. Ude , V. Tripathi , J. M. Buyck , S. Söderholm , O. Cunrath , J. Fanous , B. Claudi , A. Egli , C. Schleberger , S. Hiller , D. Bumann , Proc. Natl. Acad. Sci. U. S. A. 2021, 118, e2107644118. 34326266 10.1073/pnas.2107644118 PMC8346889

[62] G. Ramachandran , Virulence 2014, 5, 213. 24193365 10.4161/viru.27024 PMC3916377

[63] R. S. Gabarin , M. Li , P. A. Zimmel , J. C. Marshall , Y. Li , H. Zhang , J. Innate Immun. 2021, 13, 323. 34004605 10.1159/000515740 PMC8613564

[64] B. Bushnell , J. Rood , E. Singer , PLoS One 2017, 12, e0185056. 29073143 10.1371/journal.pone.0185056 PMC5657622

[65] B. J. Haas , A. Papanicolaou , M. Yassour , M. Grabherr , P. D. Blood , J. Bowden , M. B. Couger , D. Eccles , B. O. Li , M. Lieber , M. D. MacManes , Nat. Protoc. 2013, 8, 1494. 23845962 10.1038/nprot.2013.084 PMC3875132

[66] B. Langmead , S. L. Salzberg , Nat. Methods 2012, 9, 357. 22388286 10.1038/nmeth.1923 PMC3322381

[67] L. Fu , B. Niu , Z. Zhu , S. Wu , W. Li , Bioinformatics 2012, 28, 3150. 23060610 10.1093/bioinformatics/bts565 PMC3516142

[68] W. G. Yoo , J. H. Lee , Y. Shin , J. Y. Shim , M. Jung , B. C. Kang , J. Oh , J. Seong , H. K. Lee , H. S. Kong , K. D. Song , Funct. Integr. Genomics 2014, 14, 275. 24652097 10.1007/s10142-014-0366-3

[69] H. X. Dang , C. B. Lawrence , Bioinformatics 2014, 30, 1120. 24403538 10.1093/bioinformatics/btu004 PMC3982160

[70] U. Gawde , S. Chakraborty , F. H. Waghu , R. S. Barai , A. Khanderkar , R. Indraguru , T. Shirsat , S. Idicula‐Thomas , Nucleic Acids Res. 2023, 51, D377. 36370097 10.1093/nar/gkac933 PMC9825550

[71] T. Yan , D. Yoo , T. Z. Berardini , L. A. Mueller , D. C. Weems , S. Weng , J. M. Cherry , S. Y. Rhee , Nucleic Acids Res. 2005, 33, W262. 15980466 10.1093/nar/gki368 PMC1160129

[72] L. R. McLean , K. A. Hagaman , T. J. Owen , J. L. Krstenansky , Biochemistry 1991, 30, 31. 1988028 10.1021/bi00215a005

