# Supplementary material for: Novel Leech Antimicrobial Peptides, Hirunipins: Real‐Time 3D Monitoring of Antimicrobial and Antibiofilm Mechanisms Using Optical Diffraction Tomography
Source: Adv Sci (Weinh). 2025 Jan 10;12(10):2409803. doi: 10.1002/advs.202409803 (PMC11905058; doi:10.1002/advs.202409803)
Supplement: Supplementary file 1 — Supporting Information [file ADVS-12-2409803-s001.docx]

**Novel Leech Antimicrobial Peptides, Hirunipins: Real-time 3D Monitoring of Antimicrobial and Anti-biofilm Mechanisms Using Optical Diffraction Tomography**

**CONTENTS**

Microbial Strains and Cell Lines .................................................................................................S4

Minimal Inhibitory Concentrations (MICs).................................................................................S4

Hemolysis and Cytotoxicity Assays.............................................................................................S5

Membrane Depolarization Assay..................................................................................................S5

Outer and Inner Membrane Permeabilization Assay....................................................................S6

Flow Cytometry Analysis..............................................................................................................S7

SEM Imaging.................................................................................................................................S7

MBEC (minimal biofilm eradication concentration) ....................................................................S8

Confocal laser scanning microscopy (CLSM) ..............................................................................S8

Synergy testing by checkerboard assay.........................................................................................S9

Time Killing Assay........................................................................................................................S9

Measurement of TNF-α, IL-6, and MCP-1 Release from LPS-Stimulated RAW264.7 Cells...............................................................................................................................................S9

Reverse-Transcription Polymerase Chain Reaction (RT-PCR) .................................................S10

ODT-HTS Analysis for Bacterial cells .......................................................................................S11

**Supplementary Tables**

**Table S1.** Characterization of synthetic putative twenty antimicrobial peptides determined by MALDI-TOF MS..........................................................................................................................S13

**Table S2.** Summaries mRNAs libraries sequenced with Illumina platforms and processed for contaminations. ..........................................................................................................................S14

**Table S3.** Mean residual ellipticity at 222 nm ([θ]_222_) and percent α-helical contents of hirunipin

peptides in aqueous buffer, 50% TFE and 30mM SDS...............................................................S15

**Table S4.** The IC_50_ (μg/mL) of hirunipin peptides against RAW264.7, NIH-3T3 and HaCat cells

.....................................................................................................................................................S16

**Supplementary Figures**

**Fig. S1.** MALDI-TOF-MS of synthetic LP1~LP19....................................................................S17-21

**Fig. S2.** Species distributions plot from sequence mapped to uniport database ........................S22

**Fig. S3.** BUSCO completeness score of the denovo transcriptome of *Hirudo nipponica*..........S23

**Fig. S4.** Tertiary structure of hirunipin peptides predicted by automated I-TASSER server......S24

**Fig. S5.** CD spectra of hirunipin peptides in aqueous buffer, 50% TFE and 30 mM SDS..........S25

**Fig. S6**. Analytical RP-HPLC profiles and retention time (min) of synthetic hirunipin 1, 2 and 3........S26

**Fig. S7**. Dose-dependent lysis of sheep red blood cells (sRBCs) in the presence of hirunipin peptides and melittin ...................................................................................................................S27

**Fig S8.** Cell viability of RAW 264.7 (a), NIH-3T3 (b) and HaCat cells (c) in the presence of hirunipin peptides and melittin....................................................................................................S28

**Fig. S9.**  Real-time ODT imaging of biofilm formation after AMP treatment ..........................S29

**Fig S10.** Propensity of resistance development for hirunipin 2, tetracycline and ciprofloxacin against *E. coli* (KCTC 1682) ......................................................................................................S30

**Fig S11.** Effects of Hirunipin 2 on the production of pro-inflammatory cytokines in LPS-stimulated RAW 264.7 macrophages...............................................................................................................S31

**Microbial Strains and Cell Lines:** Multiple bacterial strains were used in this study. Standard stains included *Escherichia coli* (KCTC 1682), *Pseudomonas aeruginosa* (KCTC 1637), *Staphylococcus aureus* (KCTC1621) and *Staphylococcus epidermidis* (KCTC 1917) supplied from the Korean Collection for Type Cultures (KCTC) of the Korea Research Institute of Bioscience and Biotechnology (KRIBB). Clinically isolated multidrug-resistant bacteria included multidrug-resistant *Klebsiella pneumonia* (MDRKP 328-83), multidrug-resistant *Acinetobacter baumannii* (MDRAB 329-53), multidrug-resistant *Pseudomonas aeruginos*a (MDRPA 314-69), multidrug-resistant *Enterobacter cloacae* (MDRE 328-84) and multidrug-resistant *Escherichia coli* (MDREC 329-66) provided from Hospital of Chosun University, Korea. All bacterial strains were stored in 20% glycerol at −80ºC. For the subsequent experiments, the bacteria were recovered and cultured in the corresponding enrichment broth at 37ºC. The mouse macrophage RAW264.7, mouse fibroblast NIH-3T3 and human keratinocyte HaCat cells were cultured in Dulbecco’s modified Eagle’s medium (DMEM), supplemented with 10% (v/v) of fetal bovine serum (Sigma-Aldrich), 100 U/mL of penicillin and 0.1 mg/mL of streptomycin, at 37ºC in the presence of 5% CO_2_

**Minimal Inhibitory Concentrations (MICs):** Bacteria were grown to mid-logarithmic phase and diluted in Mueller-Hinton broth (MHB) (Difco). The bacterial suspension (1×10^6^ CFU/well) was added to a 96-well microtiter plate along with a two-fold serial dilution of samples. The plate was incubated at 37 °C for 24 hours and the bacterial growth inhibition was assessed by measuring the optical density using an ELISA plate reader. The minimum inhibitory concentration (MIC) was defined as the lowest concentration of samples that prevented visible bacterial growth.

**Hemolysis and Cytotoxicity Assays:** The hemolytic activity of hirunipin peptides was determined using sheep red blood cells (sRBCs). The sheep erythrocytes were diluted in PBS to a final concentration of 4% (v/ v). Then, 100 μL of peptide solutions (1−128 μM) were added to a 96-well plate containing 100 μL of the erythrocyte suspension. After 1 hour of incubation at 37°C and subsequent centrifugation at 1000g for 10 min, 100 μL of supernatants was transferred to another 96-well plate. The released hemoglobin was determined using a microplate reader at 450 nm. Percent hemolysis was calculated by the following formula: Percentage hemolysis = 100 × [(A − A_0_) / (A_t_ − A_0_)] where A is the absorbance of the peptide sample at 540 nm and A_0_ and A_t_ are zero percent and 100% hemolysis in PBS and 0.1% Triton X-100, respectively. The cytotoxicity of hirunipin peptides toward mouse macrophage RAW264.7, mouse fibroblast NIH-3T3 and human keratinocyte HaCat cells was determined by MTT method [32,33]. RAW264.7, NIH-3T3 and HaCat cells were plated into 96-well plates at a density of 10000 cells per well in 100 mL of medium and grown for overnight. The cells were then exposed to peptides at different concentrations (1−64 μg/mL) for 48 hours. Then, 100 μL of MTT solution (0.5 mg/mL) was added to each well. After further incubation for another 4 hours, formazan formed from MTT was extracted in 150 μL of DMSO for 15 min of standby. The absorbance at 550 nm was then determined on a microplate reader. Percent cell viability was calculated with the following formula: Percent cell viability = [(A − A_0_)/(A_t_ − A_0_)] × 100%, here, A, A_0_, and A_t_ represent the absorbance of the peptide, DMSO, and control, respectively.

**Membrane Depolarization Assay:** Briefly, mid-log phase *S. aureus* (KCTC 1621) was harvested and washed three times with 5 mM HEPES buffer (pH 7.4, containing 20 mM glucose), and resuspended to an OD_600_ of 0.05 in the same buffer. The cell suspension was incubated with 0.4 μM diSC_3_-5 for 90 min such that most of the dye molecules gathered at the cytoplasmic membrane. To equilibrate the cytoplasmic and external K^+^ concentrations, KCl was added to the cell suspension containing diSC_3_-5 to obtain a final concentration of 100 mM, followed by incubating the cells at room temperature for 15−30 min, with peptide aliquots added to 2 mL of suspension. The fluorescence was recorded using a model RF-5301 PC fluorescence spectrophotometer (Shimadzu) at an excitation wavelength of 622 nm and an emission wavelength of 670 nm.

**Outer and Inner Membrane Permeabilization Assay:** Changes in the integrities of outer membrane (OM) of *E. coli* (KCTC 1682) caused by the presence of hirunipin peptides were determined by the uptake of N-phenylnaphthalen-1-amine (NPN), a fluorescent dye that is sensitive to the outer membrane, as reported previously [32,33]. To prepare the bacterial suspension, *E. coli* KCTC 1682 cells were grown to mid-log phase and washed three times in 5 mM HEPES buffer (pH 7.4) containing 20 mM glucose and 5 mM KCN. The cells were then diluted to an optical density of 0.05 at 600 nm in the same buffer. NPN was dissolved in acetone to make a 1 mM stock solution, and 30 μL of this solution was added to the bacterial suspension to obtain a final concentration of 10 μM. The fluorescence of NPN (excitation λ = 350 nm, emission λ = 420 nm) was measured until it reached a stable level. Then, different concentrations of peptides were added to the suspension and the fluorescence was monitored over time until no further increase was observed. The inner membrane permeabilization of *E. coli* ML-35 in the presence of hirunipin peptides was assessed through the measurement of β-galactosidase activity using ONPG (o-nitrophenyl-β-D-galactoside), a substrate for cytoplasmic β-galactosidase, as described previously [32,33]. *E. coli* ML-35 cells were grown to mid-log phase and resuspended in sample buffer (10 mM sodium phosphate, 100 mM NaCl, pH 7.4) containing 1.5 mM ONPG to an optical density of 0.5 at 600 nm. The effect of peptides on the inner membrane permeability was evaluated by measuring the absorbance at 405 nm. The absorbance increase reflects the hydrolysis of ONPG to o-nitrophenol, which is a yellow compound. ONPG can enter the cells through the permeabilized inner membrane and be cleaved by the cytoplasmic enzyme β-galactosidase.

**Flow Cytometry Analysis:** *E. coli* (KCTC 1682) and *S. aureus* (KCTC 1621) were cultured in LB broth until they reached mid-log-phase, then washed and resuspended in PBS to a density of 2 × 10^5^ CFU/mL. The bacterial suspension was incubated with the samples and 10 μg/mL of propidium iodide (PI), a fluorescent dye, at 37ºC for 1 hour in the dark. The samples were then centrifuged and washed twice in PBS to remove any excess dye. The fluorescence of PI, which indicates membrane damage, was measured by FACS flow cytometer (Agilent, ACEA Bioscience Inc.) using a laser excitation wavelength of 488 nm.

**SEM Imaging:** Membrane morphological changes of hirunipin peptide-treated *E. coli* (KCTC 1682) were visualized by employing SEM, as described earlier [32,33]. *E. coli* (KCTC 1682) cells were grown to mid-log-phase (OD_600_ = 0.1) and exposed to peptides (1×MIC or 2×MIC) for 4 hours. The cells were then collected and fixed overnight with 2.5% (w/v) glutaraldehyde at 4°C. After fixation, the cells were dehydrated in a series of ethanol solutions (50, 70, 90, and 100%) for 10 min each, followed by a mixture of ethanol and tert-butanol (1:1, v/v) for 15 min, and pure tert-butanol for 15 min. The samples were dried using liquid CO_2_ in a critical point dryer, coated with gold−palladium, and observed by scanning electron microscopy (Zeiss Gemini 500 Field).

**MBEC (minimal biofilm eradication concentration):** The biofilm eradication concentration (MBEC) of the peptides against MDRAB (329-53) strain was determined using the Calgary Biofilm Device (CBD) from Innovotech [32,33]. A bacterial suspension of 1 × 10^6^ CFU/mL in 150 μL of Luria-Bertani (LB) media was added to each well of a 96-well microtiter plate with a peg lid (Innovotech, product code: 19111). The plate was incubated at 37°C for 24 hours with shaking at 110 rpm to form biofilms on the pegs. The peg lid was rinsed with phosphate-buffered saline (PBS, 0.01 M) and transferred to a new plate containing different concentrations of the peptides (200 μL per well). The plate was incubated at 37°C for 24 hours with shaking at 110 rpm to expose the biofilms to the peptides. The peg lid was rinsed again with PBS and transferred to a recovery plate with 200 μL of LB media per well. The recovery plate was sonicated in a water bath for 10–15 min to detach the biofilms from the pegs. The recovery plate was incubated at 37°C for 24 hours with shaking at 110 rpm to allow the surviving bacteria to grow and produce turbidity. The MBEC was defined as the lowest peptide concentration that prevented turbidity in the recovery plate compared to the sterility controls. The experiment was repeated three times and the median value of each trial was reported.

**Confocal laser scanning microscopy (CLSM):** MDRAB (329-53) cells (1×10^6^ CFU/mL) were inoculated in 24-well plates with Mueller-Hinton broth (MHB) and sterile discs. The plates were incubated at 37°C for 24 hours to allow biofilm formation on the discs. The discs were washed three times with phosphate-buffered saline (PBS) to remove planktonic cells and transferred to new plates with MHB and the peptides (64 or 128 μg/mL). The plates were incubated at 37°C for 6 hours to expose the biofilms to the peptides. The discs were washed twice with PBS and stained with a mixture of SYTO 9 (6.7 μM) and propidium iodide (PI, 40 μM) for 30 min in the dark at 37°C. The biofilm structure and viability on the discs were visualized and analyzed by confocal laser scanning microscopy (CLSM) using a Zeiss LSM 710 Meta microscope and ZEN 2009 Light Edition software from ZEISS Microscopy.

**Synergy testing by checkerboard assay:** Briefly, each 96-well microtiter plate was inoculated with two-fold serial dilutions of two antibiotics, ranging from 1/8 MIC to 2 MIC, such that one antibiotic was inoculated in the columns and the other in the rows. Then, MDRAB (329-53) cells were inoculated at a concentration of 1× 10^6^ CFU/mL. The plates were incubated overnight under static conditions and observed visually. The fractional inhibitory concentration (FIC) index (FICI) was calculated as follows: FICI = [(MIC of antibiotic in combination) / (MIC of antibiotic alone)] + [(MIC of small molecule in combination) / (MIC of small molecule alone)], where FICI ≤ 0.5 indicates synergism, 0.5 < FICI ≤ 1.0 indicates an additive effect, 1.0 < FICI ≤ 4.0 represents indifference, and FICI > 4.0 shows antagonism.

**Time Killing Assay***:* MDRAB (329-53) suspension (1 × 10^6^ CFU/mL) was added to hirupinin 2 and antibiotic alone or in combination at synergistic concentration in an equal volume. Aliquots (5 μL) were taken periodically at different time points and were diluted before being cultured in LB solid medium for 24 hours. The number of colonies on the LB solid medium was counted. Each test was performed three times at least, and the average value was reported.

**Measurement of TNF-α, IL-6, and MCP-1 Release from LPS-Stimulated RAW264.7 Cells:** RAW264.7 murine macrophage cells (5×10^5^ cells/mL) were plated and adhered to a 96-well plate (100 μL/well). These cells were then stimulated with LPS from *Escherichia coli* O111:B4 (100 ng/mL) in the presence or absence of a peptide for 24 hours. The release of TNF-α, IL-6, and MCP-1 was quantified using a commercial ELISA kit (R&D Systems) following the manufacturer’s protocol.

**Reverse-Transcription Polymerase Chain Reaction (RT-PCR):** RAW264.7 cells were plated at a concentration of 5×10^5^ cells/well in six-well plates and incubated overnight. Hirunipin 2 was added to the wells at the concentrations of 8, 16, and 32 μg/mL. Cells intended for TNF-α, IL-6, and MCP-1 quantification were treated for 6 hours with or without (negative control) 20 ng/mL LPS, in the presence or absence of peptide, in DMEM supplemented with 10% bovine serum. The cells were detached from the wells and washed once with PBS. Total RNA was extracted using Trizol Reagent (Ambion). Subsequently, 1 μg of total RNA was converted to cDNA using TakaRa PrimescriptTM Reverse Transcriptase (TakaRa). The primers used were purchased from Bioneer. The cDNA products were amplified by AccuPower® PCR PreMix (Bioneer). For TNF-α (forward: 5′-CCTGTAGCCCACGTCGTAGC-3′; reverse: 5′-TTGACCTCAGCGCTGAGTTG-3′), IL-6 (forward: 5′-ACAACCACGGCCTTCCCTACTT-3’; reverse: 5′-CACGATTTCCCAGAGAACATGTG-3′), MCP-1 (forward, 5′-ATCCCAATGAGTAGGCTGGAGAGC-3'; reverse, 5′-CAGAAGTGCTTGAGGTGGTTGTG-3') or GAPDH (forward: 5′-GACATCAAGAAGGTGGTGAA-3’; reverse: 5′-TGTCATACCAGGAAATGAGC-3′), the amplification protocol consisted of an initial denaturation step of 5 min at 94°C, followed by 30 cycles of denaturation at 94°C for 1 min, annealing at 55°C for 1.5 min, and extension at 72°C for 1 min, concluding with a final extension step of 5 min at 94°C, followed by 30 cycles of denaturation at 94°C for 1 min, annealing at 55°C for 1.5 min, and extension at 72°C for 1 min, and afterwards by a final extension step of 5 min at 72°C.

**ODT-HTS Analysis for Bacterial cells:** ODT-HTS was performed using a low-coherence HT system (HT-X1, Tomocube) capable of simultaneous time-lapse monitoring of multiple label-free bacterial cells or variable AMP treatment conditions. Equipped with a motorized 40× objective lens (NA 0.95) and a 450 nm LED illumination module with a digital micromirror device, it serves high-resolution 3D RI tomograms. This combination minimizes speckle noise while enhancing imaging clarity, making it ideal for observing thick biological specimens. The HT-X1 system features an advanced autofocus system controlled by TomoStudioX software, which digitally identifies the optimal focal plane to ensure high-quality data acquisition, during time-lapse imaging. An integrated stage-top incubator (STXG-WSKMXA22B-E, TOKAI HIT) maintains stable environmental conditions, including temperature, humidity, and CO_2_ levels, ensuring sample viability for long-term studies. The system achieves high-resolution imaging with lateral and axial resolutions of 155 nm and 947 nm, respectively. Additionally, it supports automated data acquisition and RI tomogram stitching, enabling large field-of-view (FOV) reconstructions and comprehensive analysis of dynamic biological processes. *E. coli* is prepared at 1.0 × 10^7^ cells/mL in PBS on a custom-designed 96-well plate with PI (2 μg/mL). Peptides (32 μg/mL) were added to each well. Specifically, for 96-well plates, we used OEM products manufactured by ibidi (Cat# 900105-01).

For peptide screening, ODT-HTS was used to perform quantitative imaging of bacterial cells after two hours of peptide treatment. The total number of bacterial cells was determined using cell detection algorithm in TomoAnalysis version 1.7.13 (Tomocube), which detects individual *E. coli* cells based on their RI characteristics. Since dead bacterial cells can be identified using its own RI value and increased FL signal via PI accumulation [22], the number of PI-positive cells was calculated by correlating RI values with FL signals specific to PI, using the HT-FL correlation method integrated into the TomoAnalysis version 1.7.13 (Tomocube). The proportion of PI-positive cells was then calculated as the ratio of PI-positive cells to the total cell count. This method was also applied for real-time bacterial cell counting during AMP treatment.

**Table S1.** Characterization of synthetic putative twenty antimicrobial peptides determined by MALDI-TOF-MS

| Peptides | Molecular mass (M) | |
| --- | --- | --- |
|  | Calculated (M) | Observed |
| LP1 | 1839.47 | [M+H]^+^ = 1839.1686 |
| LP2 | 2677.40 | [M+H]^+^ = 2676.2263 |
| LP3 | 3214.98 | [M+H]^+^ = 3214.6420 |
| LP4 | 4025.84 | [M+H]^+^ = 4026.8776 |
| LP5 | 2767.56 | [M+H]^+^ = 2767.7710 |
| LP6 | 3607.57 | [M+2Na]^+^ = 3657.1330 |
| LP7 | 3134.06 | [M+H]^+^ = 3134.6818 |
| LP8 | 3256.32 | [M+H]^+^ = 3256.2040 |
| LP9 | 2519.31 | [M+H]^+^ = 2519.0517 |
| LP10 | 4137.24 | [M+2Na]^+^ = 4186.2341 |
| LP11 | 2602.31 | [M+H]^+^ = 2601.6660 |
| LP12 | 3108.01 | [M+H]^+^ = 3107.4924 |
| LP13 | 2533.18 | [M+H]^+^ = 2533.4780 |
| LP14 | 2692.44 | [M+H]^+^ = 2692.4470 |
| LP15 | 4187.37 | [M+2Na]^+^ = 4233.6688 |
| LP16 | 4279.45 | [M+H]^+^ = 4280.6547 |
| LP17 | 3035.72 | [M+H]^+^ = 3036.3233 |
| LP18 | 2950.83 | [M+H]^+^ = 2950.1666 |
| LP19 | 3495.41 | [M+Na]^+^ = 3520.0494 |

**Table S2.** Summaries of mRNAs libraries sequenced with Illumina platforms and processed for contaminations.

| Tissue | Names | Raw | Trimmed | Removed bacteria | Removed Small organelle |
| --- | --- | --- | --- | --- | --- |
| Head | Sample 1 | 49,296,372 | 48,678,660 | 44,650,790 | 37,217,262 |
|  | Sample 2 | 60,326,488 | 59,704,924 | 49,838,396 | 43,942,088 |
| Salivary gland | Sample 1 | 119,450,244 | 119,040,102 | 111,965,432 | 98,323,960 |
|  | Sample 2 | 82,749,340 | 81,248,126 | 53,382,176 | 43,798,350 |
| Teeth | Sample 1 | 121,067,896 | 120,579,544 | 111,098,552 | 86,202,296 |
|  | Sample 2 | 69,014,558 | 68,165,540 | 53,382,176 | 43,798,350 |

**Table S3.** Mean residual ellipticity at 222 nm ([θ]_222_) and percent α-helical contents of hirunipin peptides in aqueous buffer, 50% TFE and 30 mM SDS

| Peptide | Buffer | | 50 % TFE | | 30mM SDS | |
| --- | --- | --- | --- | --- | --- | --- |
|  | [θ]_222_ | % α-helix | [θ]_222_ | % α-helix | [θ]_222_ | % α-helix |
| Hirunipin 1 | 2598.5 | rc | -5823.2 | 8.6 | -6531.9 | 10.7 |
| Hirunipin 2 | -2589.0 | rc | -14987.6 | 36.3 | -13777.9 | 32.7 |
| Hirunipin 3 | 2437.0 | rc | -8695.1 | 17.3 | -9715.7 | 20.4 |

% α-helix = –100 ([θ]_222_ + 3000)/33000. rc means random coil.

**Table S4.** The IC_50_ (μg/mL) of hirunipin peptides against RAW264.7, NIH-3T3 and HaCat cells

| Cells | IC_50_^a^ (μg/mL) | | | |
| --- | --- | --- | --- | --- |
|  | Hirunipin 1 | Hirunipin 2 | Hirunipin 3 | Melittin |
| RAW264.7 | 45.0 | 46.0 | 60.0 | 3.7 |
| NIH-3T3 | 50.6 | 57.6 | 52.0 | 2.1 |
| HaCat | 42.0 | >64.0 | >64.0 | 6.2 |

^a^ IC_50_ was the concentration of the compound needed to inhibit cell growth by 50%.


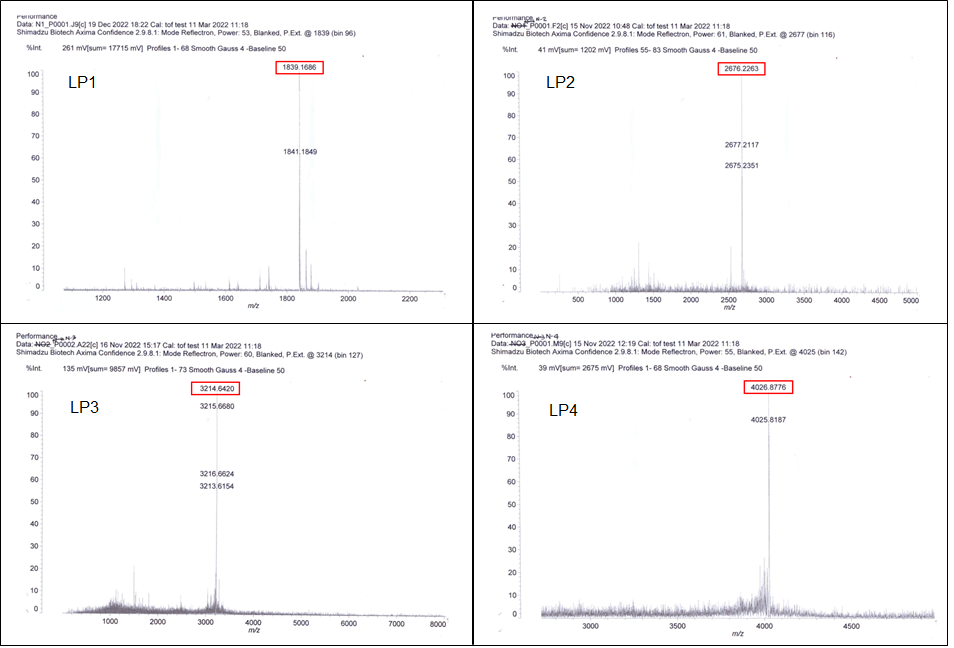


**Fig. S1**. MALDI-TOF-MS of synthetic LP1, LP2, LP3 and LP4

**
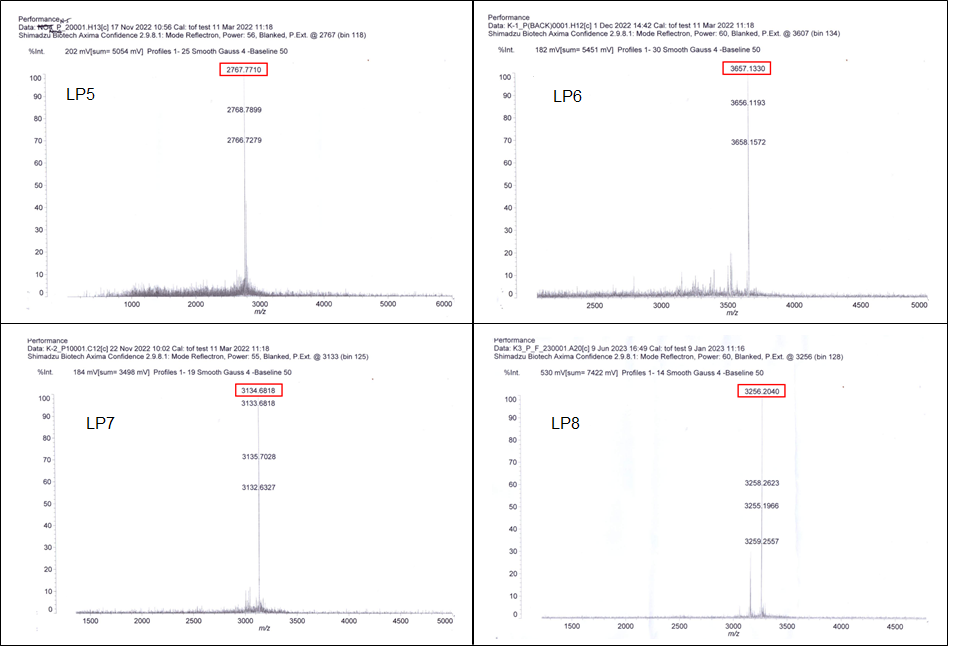
**

**Fig. S1**. MALDI-TOF-MS of synthetic LP5, LP6, LP7 and LP8


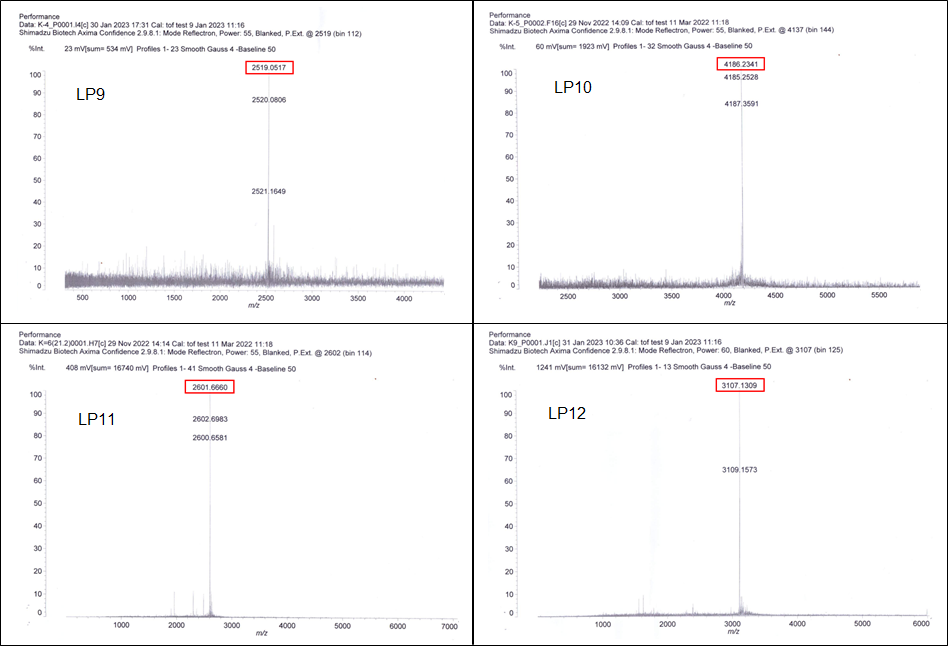


**Fig. S1**. MALDI-TOF-MS of synthetic LP9, LP10, LP11 and LP12


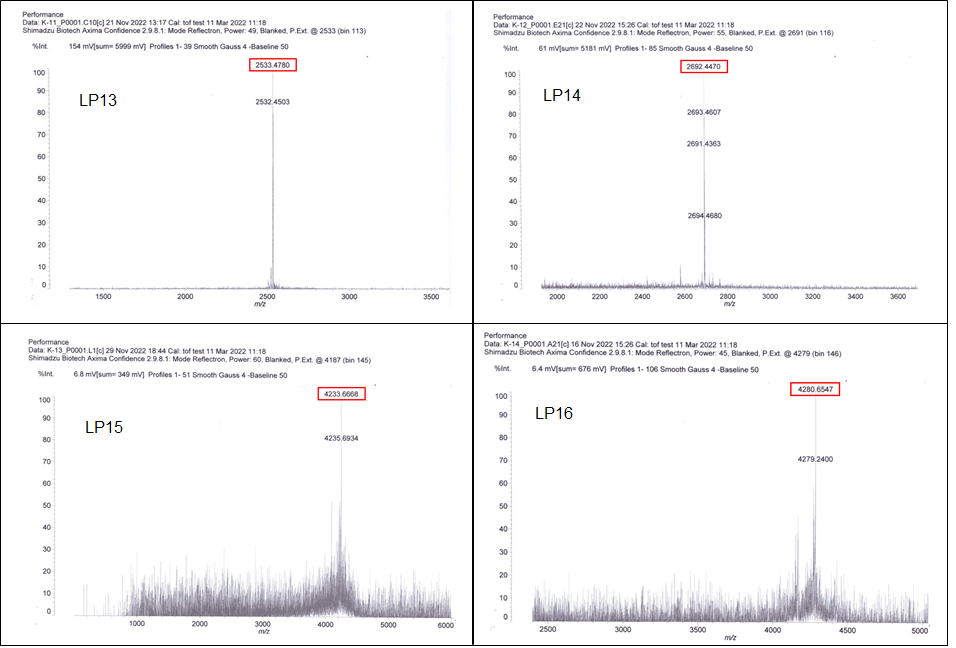


**Fig. S1**. MALDI-TOF-MS of synthetic LP13, LP14, LP15 and LP16

**
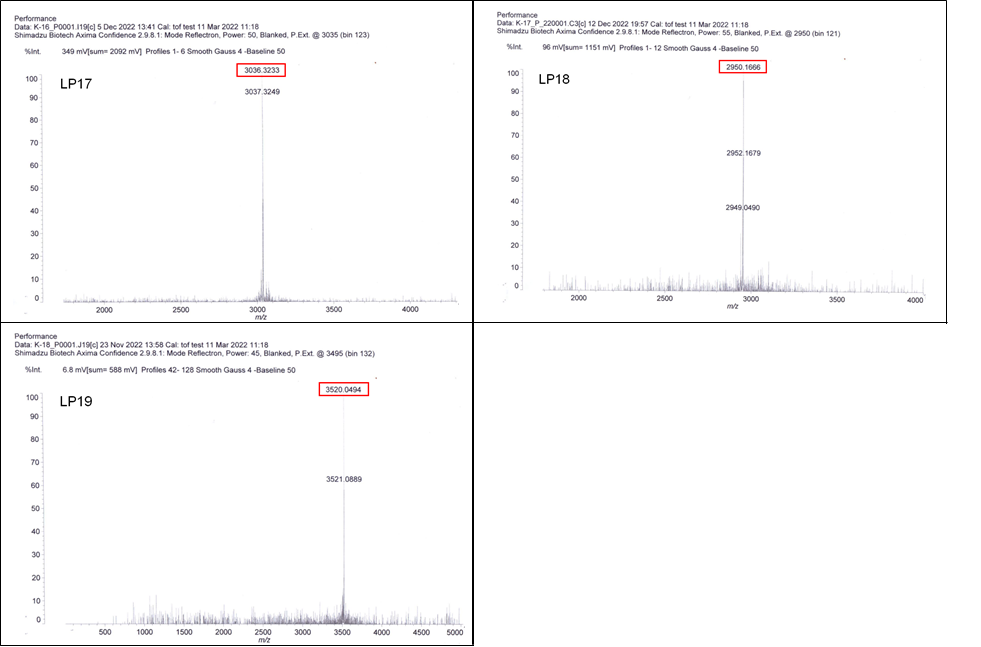
**

**Fig. S1**. MALDI-TOF-MS of synthetic LP17, LP18, and LP19

**
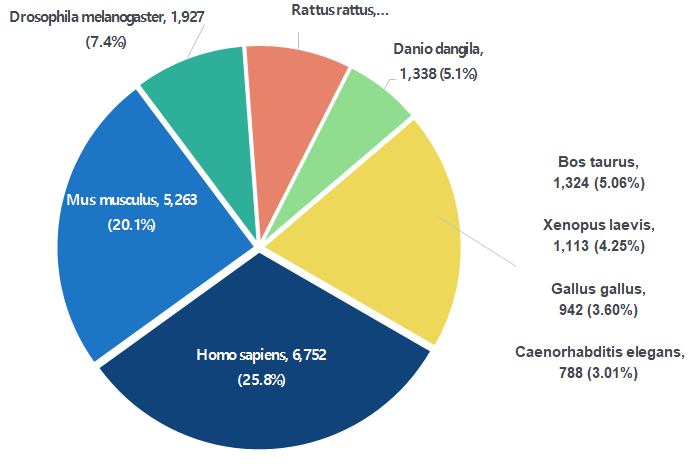
**

**Fig. S2**. Species distributions plot from sequence mapped to uniport database.

**
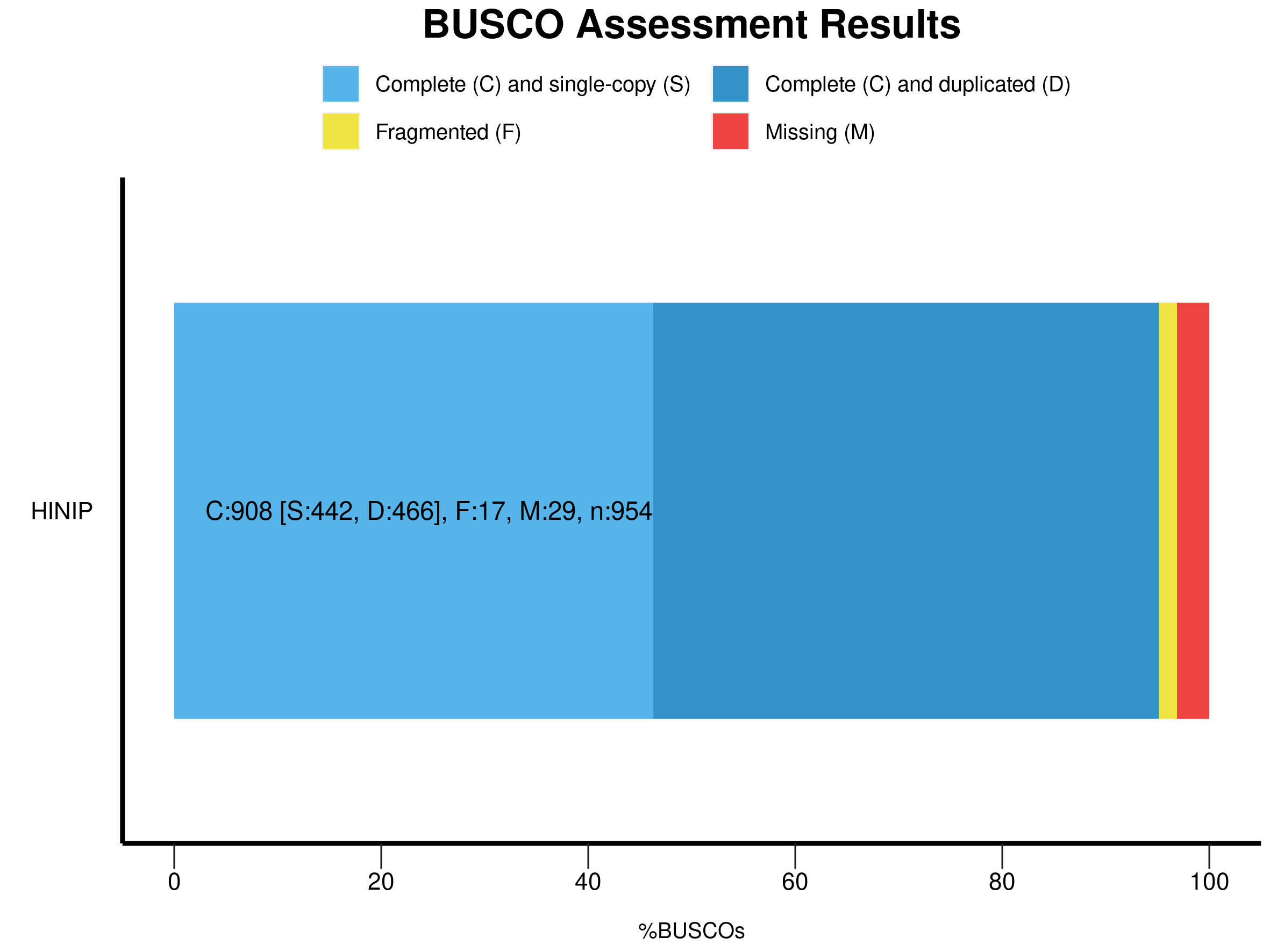
**

**Fig. S3**. BUSCO completeness score of the denovo transcriptome of *Hirudo nipponica*


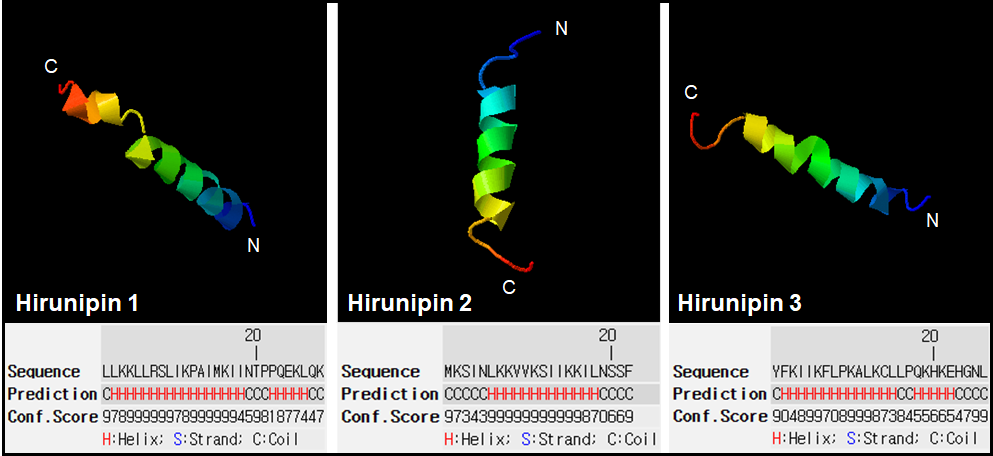


**Fig. S4.** Tertiary structure of herunipin peptides predicted by automated I-TASSER server (<http://zhanglab.ccmb.med.umich.edu/I-TASSER/)>.


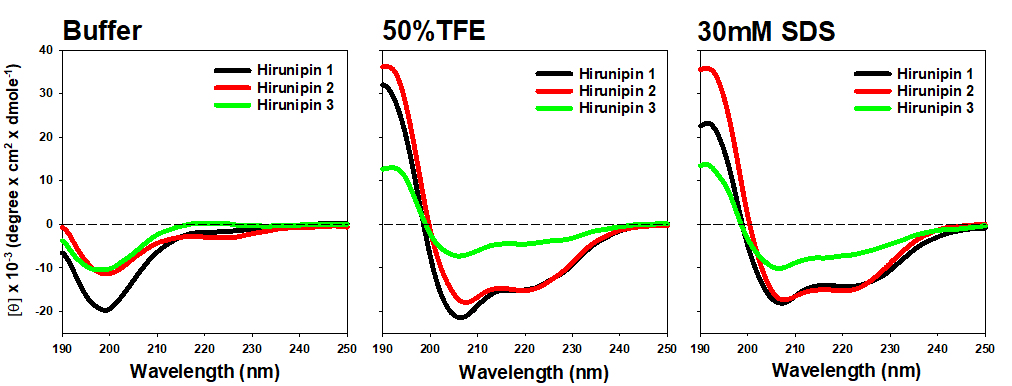


**Fig. S5.** CD spectra of hirunipin peptides in aqueous buffer, 50% TFE and 30 mM SDS


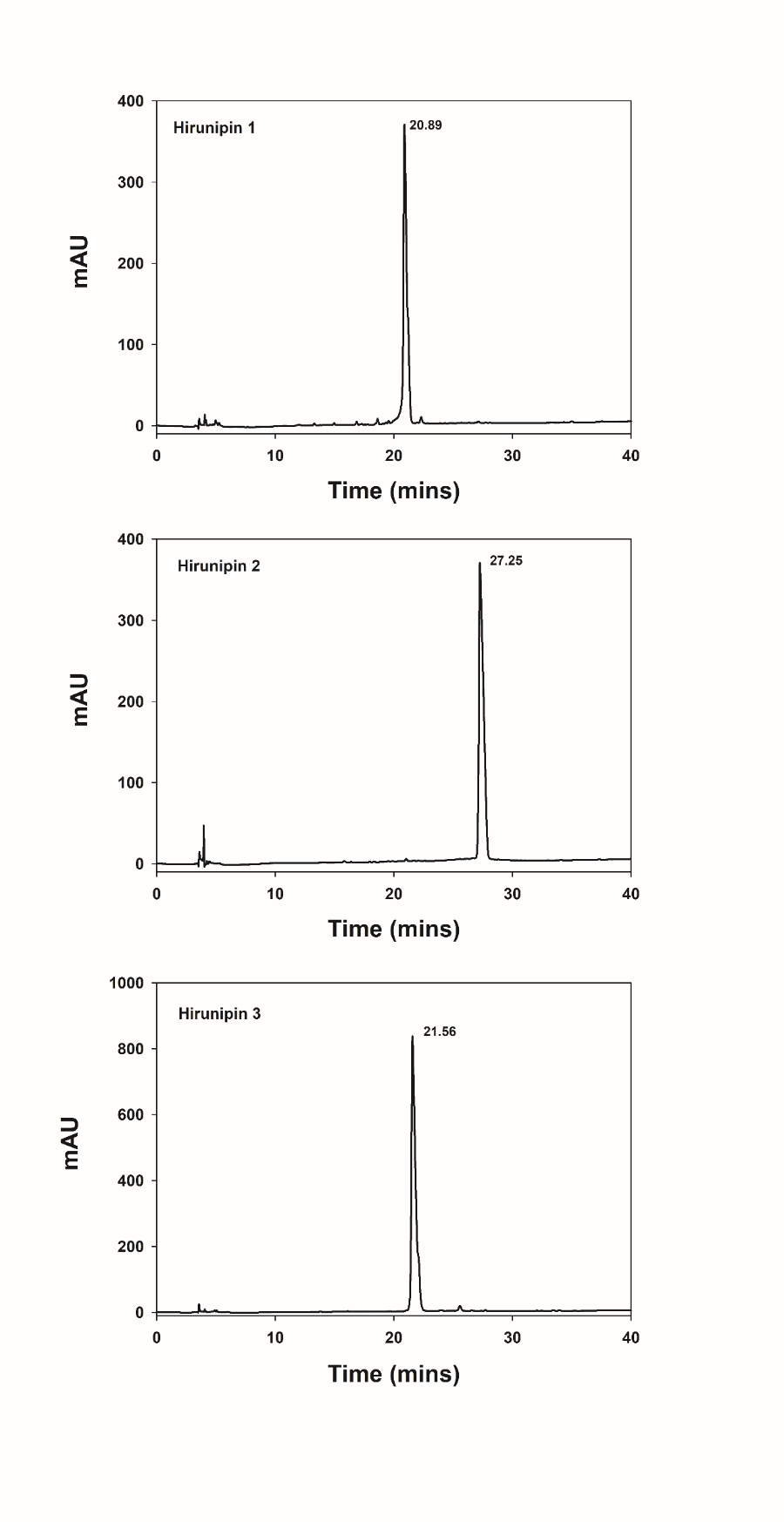


**Fig. S6**. **Analytical RP-HPLC profiles and retention time (min) of synthetic hirunipin 1, 2 and 3.** The peptides were eluted for 60 min with a flow rate of 1.0 mL/min by analytical RP-HPLC on a C_18_ column (5 mm; 4.6 mm × 250 mm;) using a gradient of buffer B (0.05% TFA in CH_3_CN/H_2_O 90:10 v/v) in buffer A (0.05 %TFA in H_2_O).

**Fig. S7.** Dose-dependent lysis of sheep red blood cells (sRBCs) in the presence of hirunipin peptides and melittin.


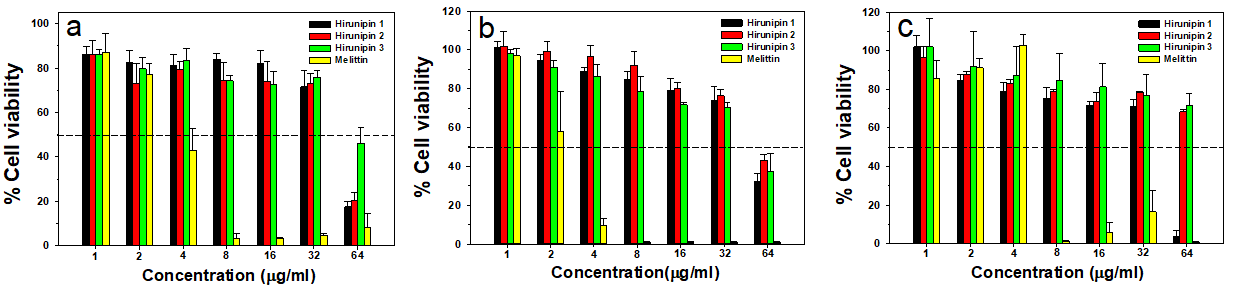
**Fig. S8.** Cell viability of RAW 264.7 (a), NIH-3T3 (b) and HaCat cells (c) in the presence of hirunipin peptides and melittin.


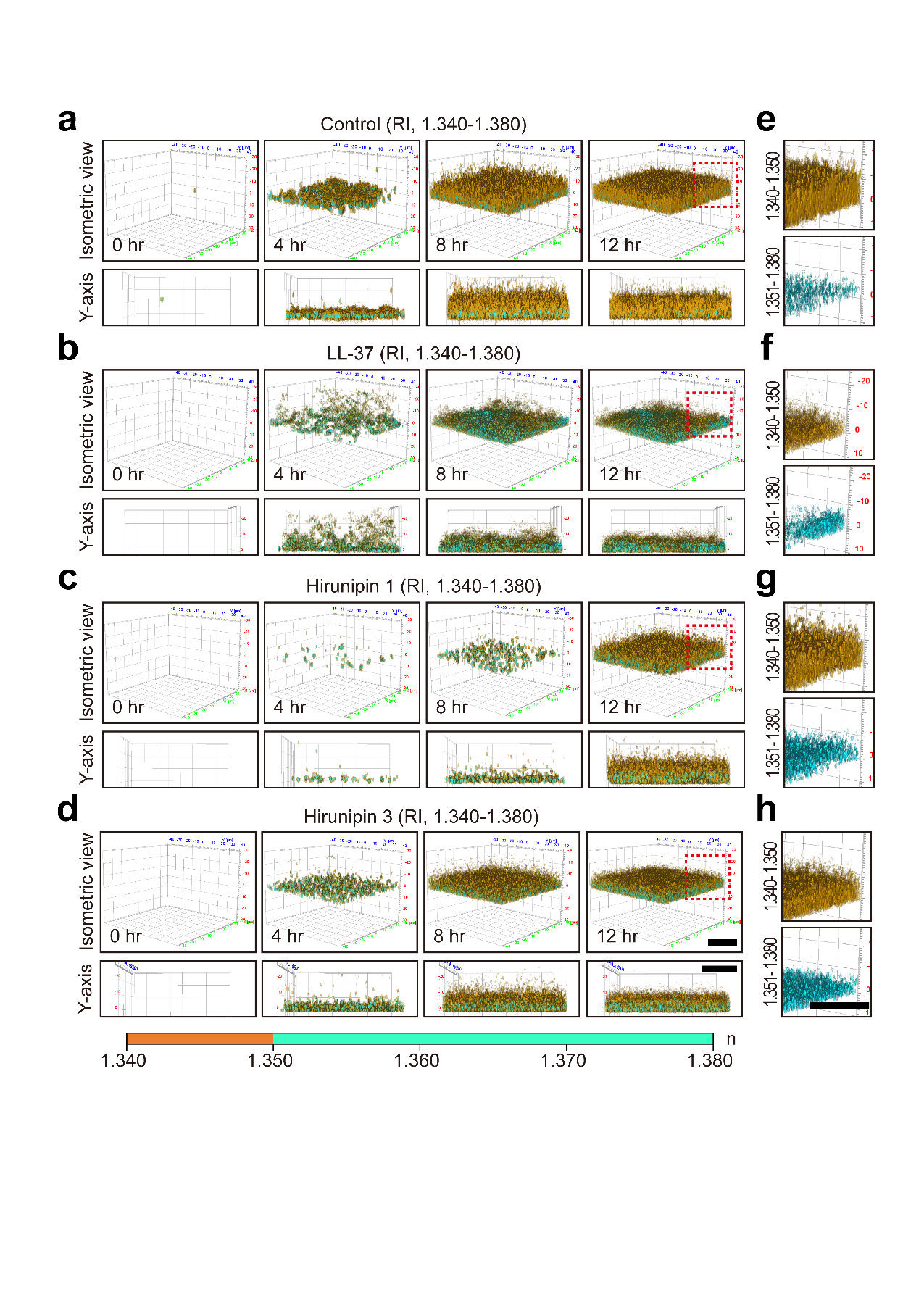


**Fig. S9.**  Real-time ODT imaging of biofilm formation after AMP treatment. **(a-d)** Representative 3D ODT images of MDRAB biofilms untreated as control **(a)** or treated with LL-37 **(b)**, hirunipin 1 **(c)**, and hirunipin 3 **(d)** over 12 h. Images for each time are shown in isometric view at the top and Y-axis view at the bottom. **(e-h)** Segmented 3D ODT images of MDRAB biofilm for control **(e)**, LL-37 **(f)**, hirunipin 1 **(g)**, and hirunipin 3 **(h)** as insets of Fig. S9a-d. Images are shown in specific RI range (top, RI = 1.340-1.350; bottom, RI = 1.351-1.380). Each color bar indicates the 3D-rendered RI distribution range (from 1.340 to 1.380). Scale bars = 30 μm.

**Fig. S10.**  Propensity of resistance development for hirunipin 2, tetracycline and ciprofloxacin against *E. coli* (KCTC 1682).

**
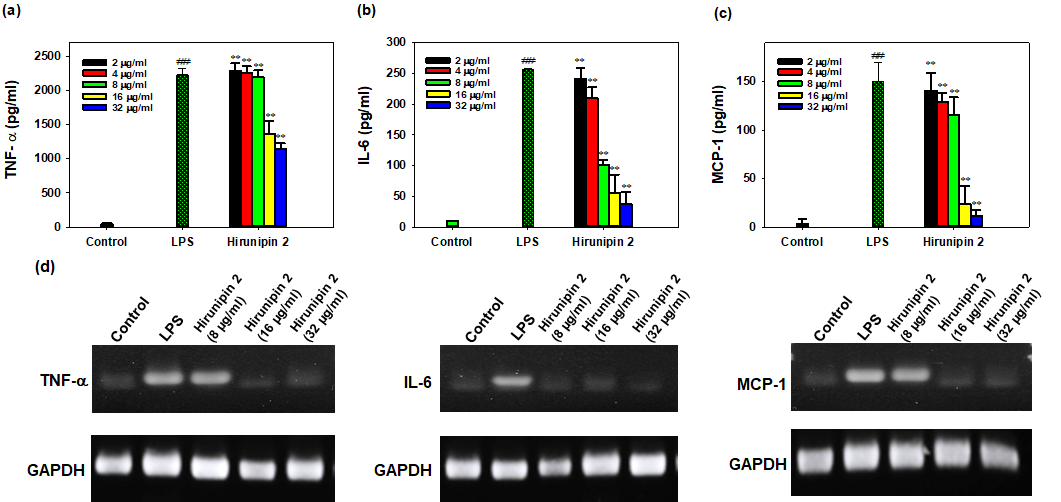
**

**Fig. S11. Effects of Hirunipin 2 on the production of pro-inflammatory cytokines in LPS-stimulated RAW 264.7 macrophages.** RAW 264.7 macrophages were exposed to LPS (20 ng/mL) in the presence and absence of varying concentrations of Hirunipin 2 for 48 hours. The levels of TNF-α (a), IL-6 (b), and MCP-1 (d) were quantified using sandwich ELISA. Data are expressed as mean ± standard deviation (SD) of multiple independent experiments. Statistical significance was determined using ## (*p < 0.01*) denoting comparison with unstimulated macrophages and ** *(p < 0.01*) indicating comparison with LPS-stimulated macrophages.
